# Supplementary material for: Skin Phototype and Disease: A Comprehensive Genetic Approach to Pigmentary Traits Pleiotropy Using PRS in the GCAT Cohort
Source: Genes (Basel). 2023 Jan 5;14(1):149. doi: 10.3390/genes14010149 (PMC9859115; doi:10.3390/genes14010149)
Supplement: Supplementary file 1 [file genes-14-00149-s001.zip › SupFigures.pdf]

Supplementary Scheme S1. Graphical abstract.

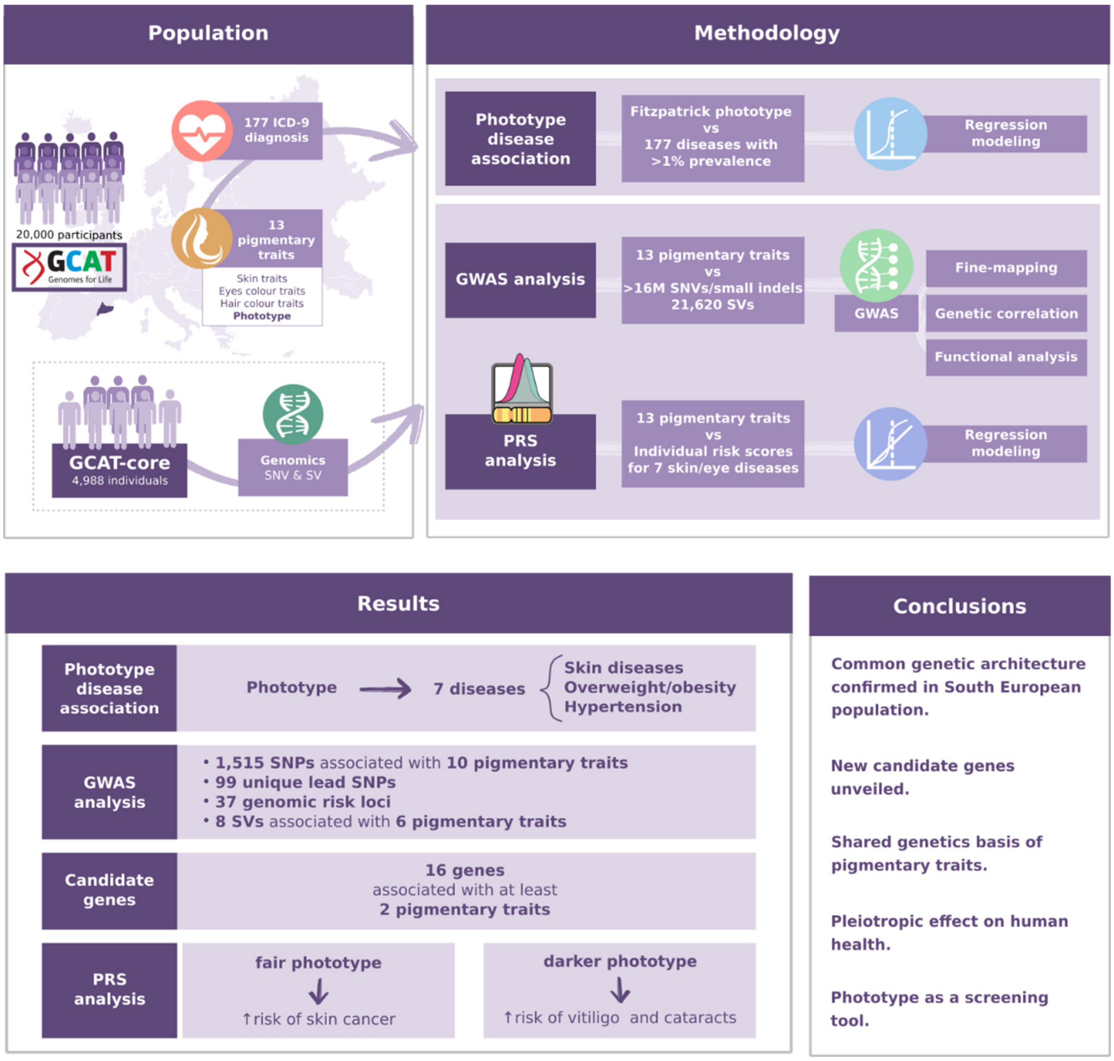

Supplementary Scheme S2. Pipeline of the study.

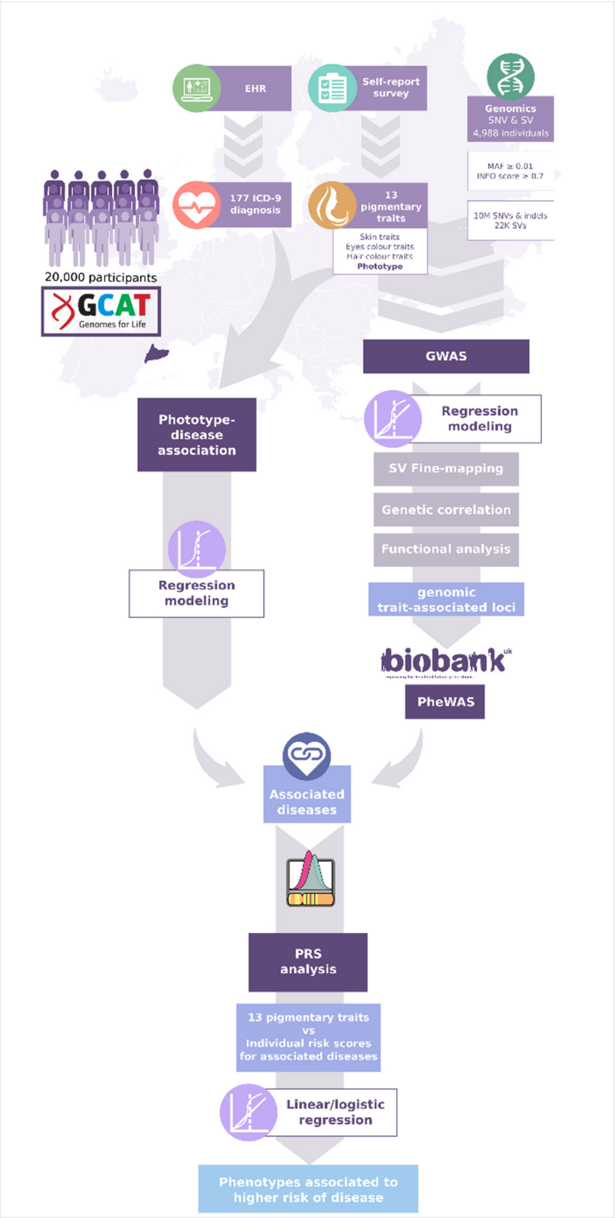

Supplementary Figure S1. Pigmentation traits distribution. Distribution of the 13 analyzed pigmentary traits in GCATcore (n = 4,988).

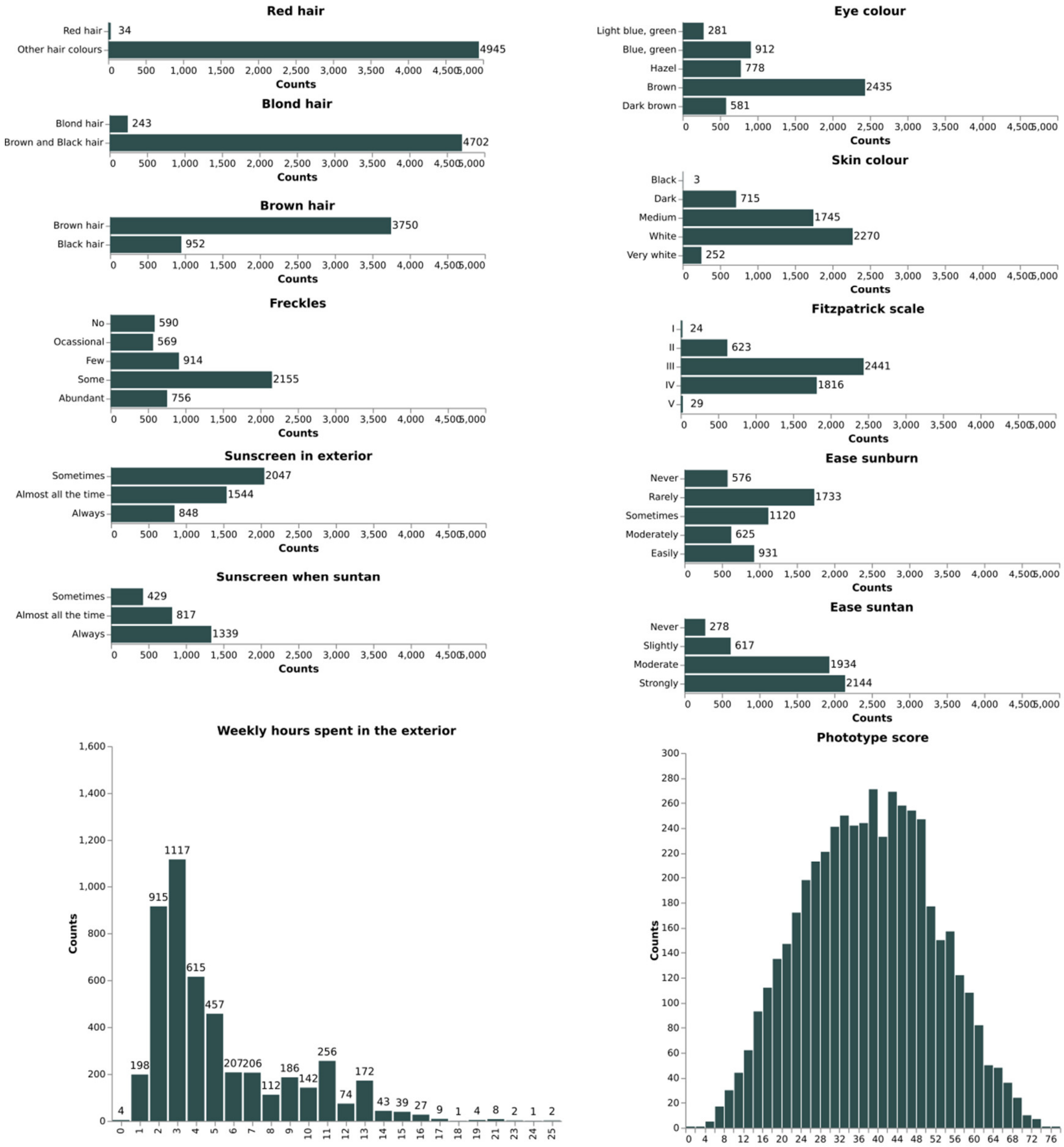

Red hair
Red hair 34
Other hair colours 4945
Counts
Blond hair
Blond hair 243
Brown and Black hair 4702
Counts
Brown hair
Brown hair 3750
Black hair 952
Counts
Freckles
No 590
Ocassional 569
Few 914
Some 2155
Abundant 756
Counts
Sunscreen in exterior
Sometimes 2047
Almost all the time 1544
Always 848
Counts
Sunscreen when suntan
Sometimes 429
Almost all the time 817
Always 1339
Counts
Eye colour
Light blue, green 281
Blue, green 912
Hazel 778
Brown 2435
Dark brown 581
Counts
Skin colour
Black 3
Dark 715
Medium 1745
White 2270
Very white 252
Counts
Fitzpatrick scale
I 24
II 623
III 2441
IV 1816
V 29
Counts
Ease sunburn
Never 576
Rarely 1733
Sometimes 1120
Moderately 625
Easily 931
Counts
Ease suntan
Never 278
Slightly 617
Moderate 1934
Strongly 2144
Counts
Weekly hours spent in the exterior
Counts
4 198 915 1117 615 457 207 206 112 186 142 256 74 172 43 39 27 9 1 4 8 2 1 2
0 1 2 3 4 5 6 7 8 9 10 11 12 13 14 15 16 17 18 19 21 23 24 25
Phototype score
Counts

Supplementary Figure S2. Phototype associated diagnosis. Significant associations between phototype (Fitzpatrick scale) and diseases after Bonferroni correction in the GCAT cohort (n = 19,205) expressed in OR and CI (95%) and ordered by p-value (lower p-value on top). Colours correspond to different ICD-9 chapters. The most significantly associated diseases were other and unspecified malignant neoplasm of skin and other dermatosis. The most represented chapter was diseases of the skin and subcutaneous tissue and neoplasms (both skin related).

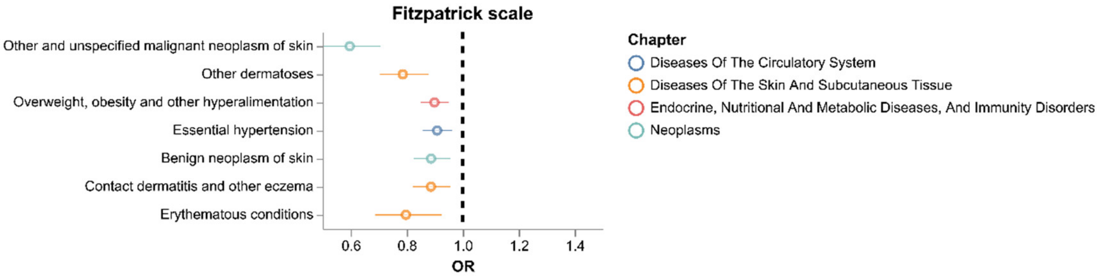

Supplementary Figure S3. Genome-wide association study plots of the 13 pigmentary traits in GCATcore (n = 4988) plotted using the generalized linear model. (A) The Manhattan plot shows the –log10 p-values (Y axis) corresponding to the location of each chromosome (X axis). Dashed horizontal lines represent the genome-wide significant (5e-8) and suggestive (1e-5) thresholds. For each significant lead SNP, rsID and nearest gene are indicated. (B) The X axis in the quartile-quartile (QQ) plot represents the expected value of the –log10 transformations, meanwhile the Y axis represents the observed value of the –log10 transformation.

A) Manhattan Plots.

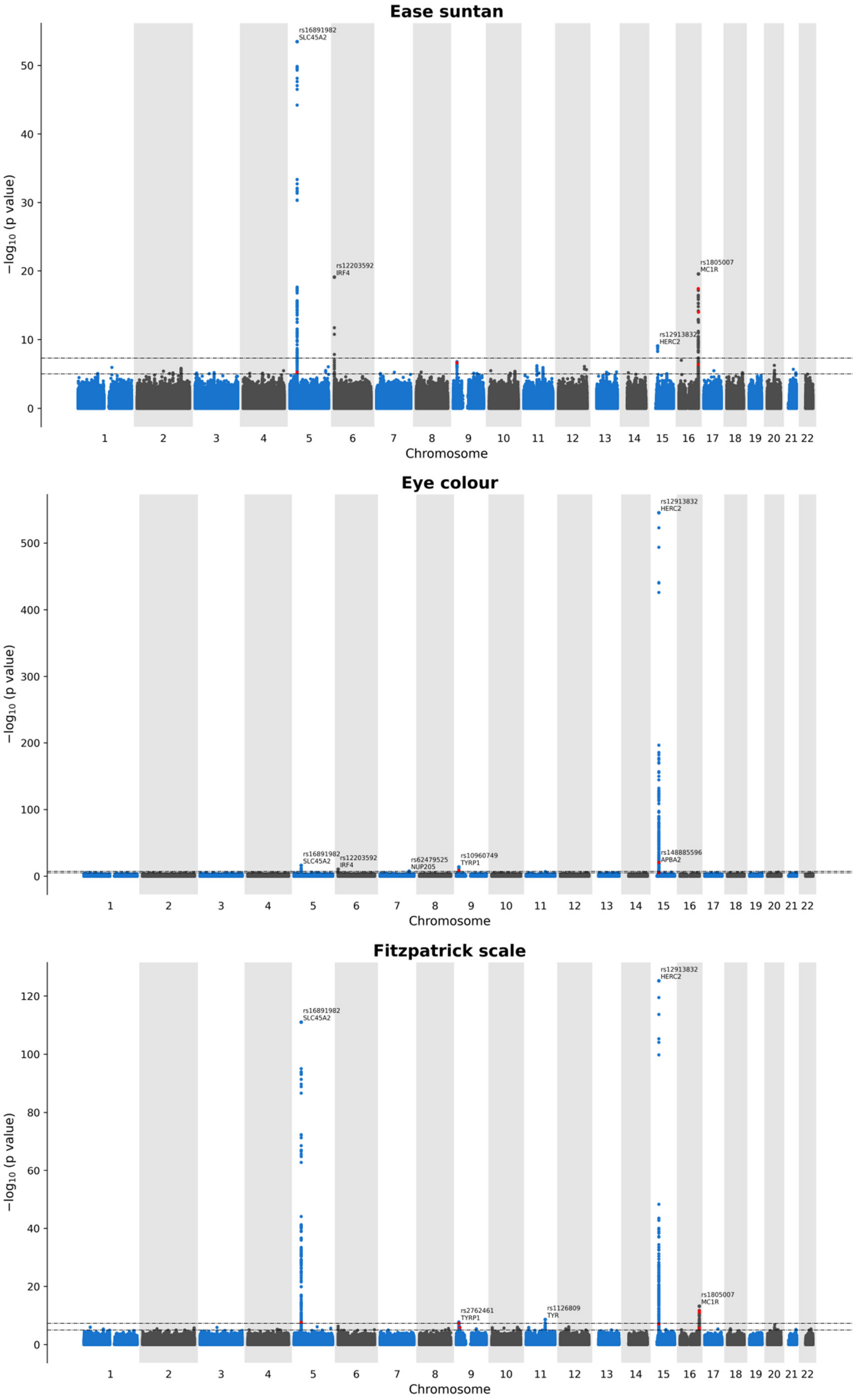
Ease suntan
−log10 (p value)
Chromosome
rs16891982
SLC45A2
rs12203592
IRF4
rs1805007
MC1R
rs12913832
HERC2
Eye colour
rs12913832
HERC2
rs16891982
SLC45A2
rs12203592
IRF4
rs62479525
NUP205
rs10960749
TYRP1
rs148885596
APBA2
Fitzpatrick scale
rs12913832
HERC2
rs16891982
SLC45A2
rs2762461
TYRP1
rs1126809
TYR
rs1805007
MC1R

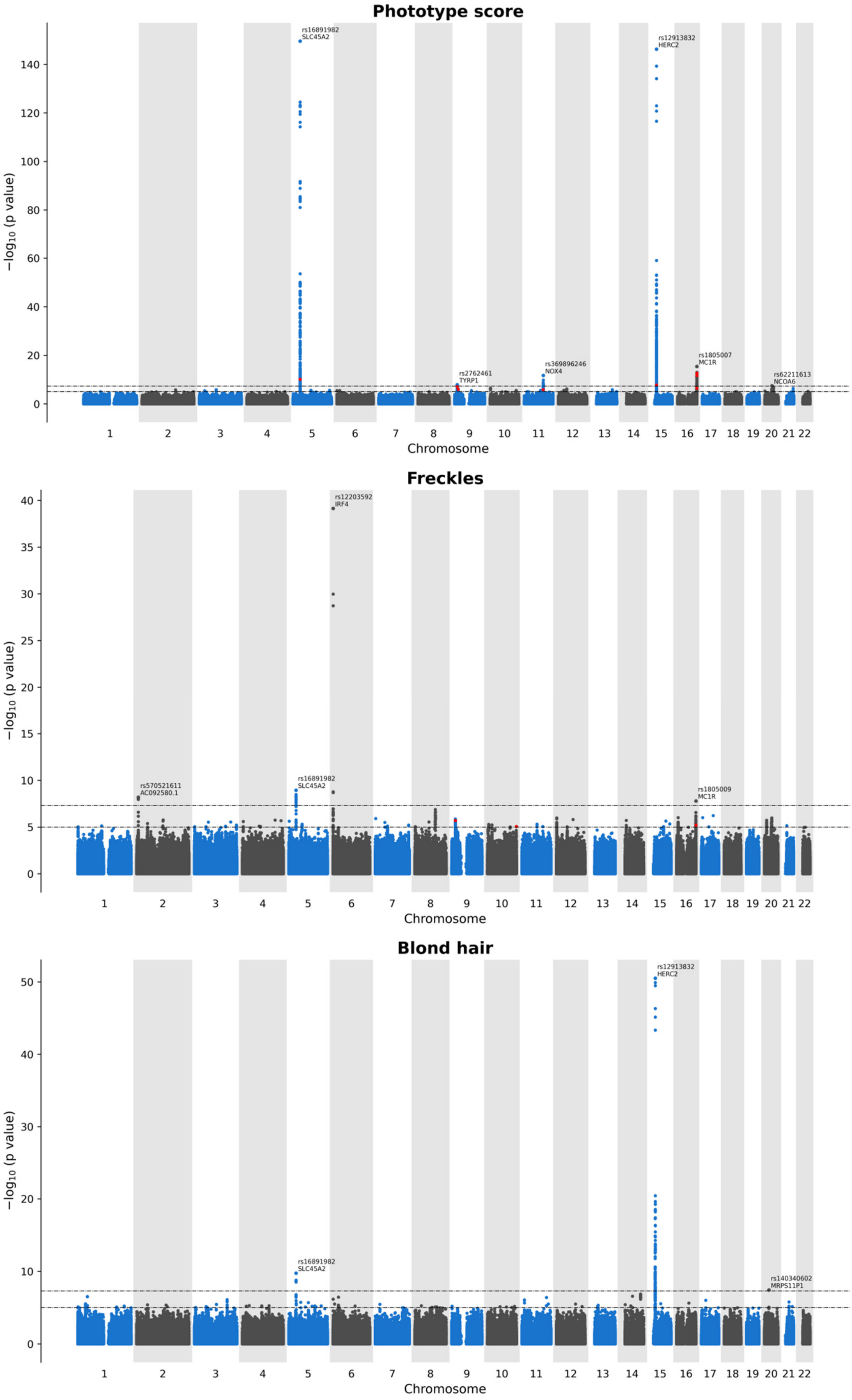
Phototype score
−log10 (p value)
Chromosome
rs16891982
SLC45A2
rs12913832
HERC2
rs2762461
TYRP1
rs369896246
NOX4
rs1805007
MC1R
rs62211613
NCOA6
Freckles
rs12203592
IRF4
rs570521611
AC092580.1
rs16891982
SLC45A2
rs1805009
MC1R
Blond hair
rs12913832
HERC2
rs16891982
SLC45A2
rs140340602
MRPS11P1

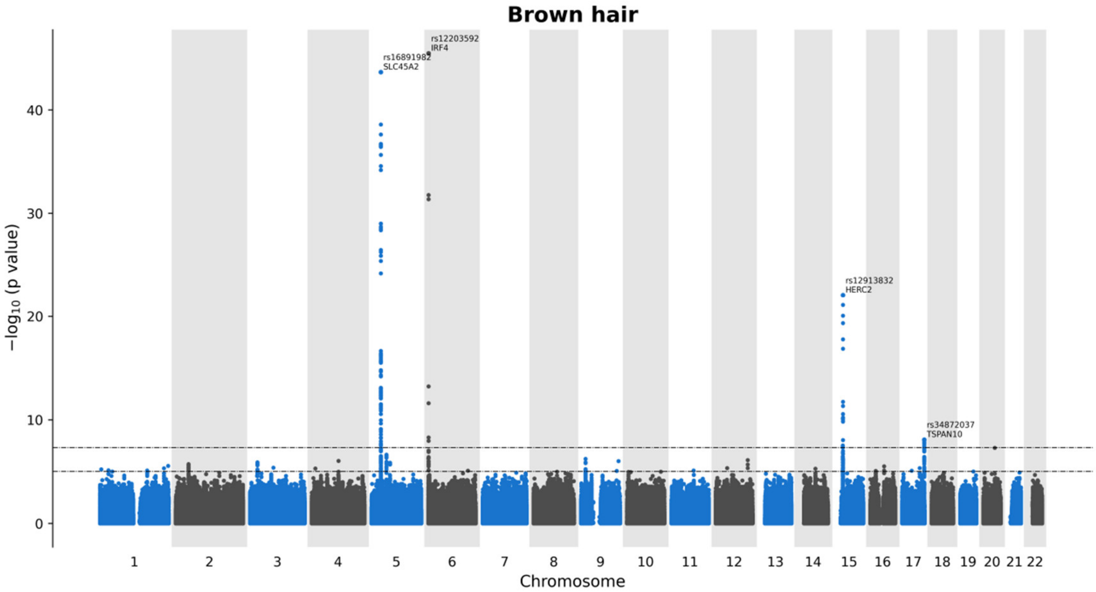

Brown hair
rs12203592
IRF4
rs16891982
SLC45A2
rs12913832
HERC2
rs34872037
TSPAN10
−log10 (p value)
40
30
20
10
0
1
2
3
4
5
6
7
8
9
10
11
12
13
14
15
16
17
18
19
20
21
22
Chromosome

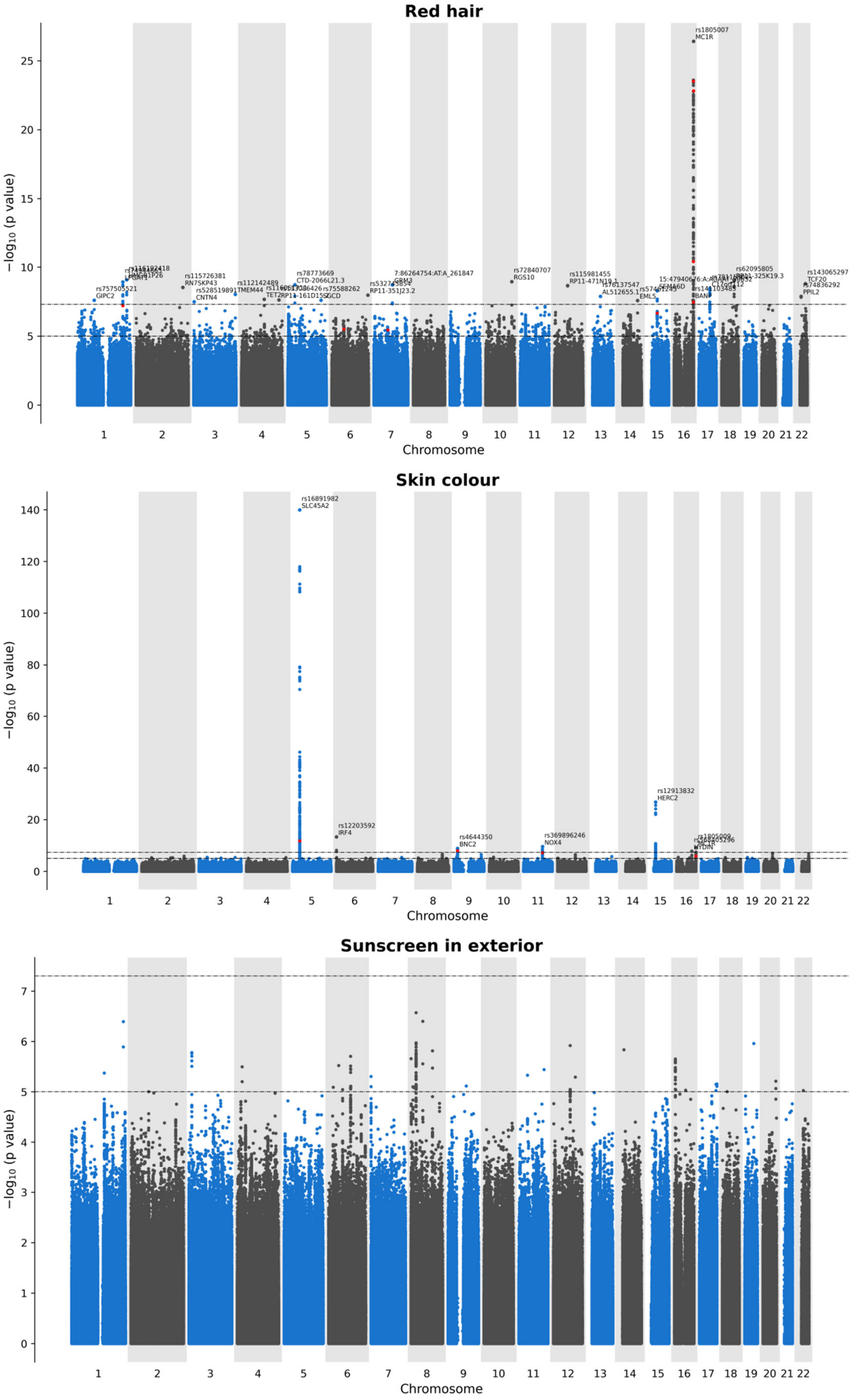

Red hair
rs1805007
MC1R
rs72840707
RGS10
rs115726381
RN7SKP43
rs78773669
CTD-2066L21.3
rs143065297
TCF20
rs74836292
PPIL2
rs62095805
−log10 (p value)
Chromosome
Skin colour
rs16891982
SLC45A2
rs12203592
IRF4
rs4644350
BNC2
rs369896246
NOX4
rs12913832
HERC2
−log10 (p value)
Chromosome
Sunscreen in exterior
−log10 (p value)
Chromosome

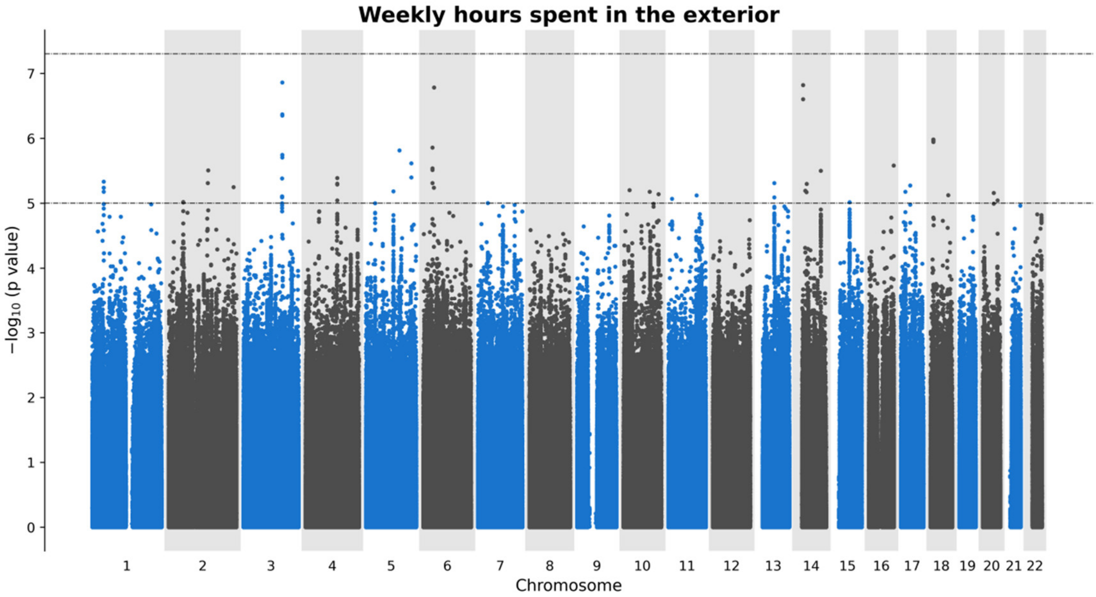
Weekly hours spent in the exterior
−log10 (p value)
Chromosome
1 2 3 4 5 6 7 8 9 10 11 12 13 14 15 16 17 18 19 20 21 22

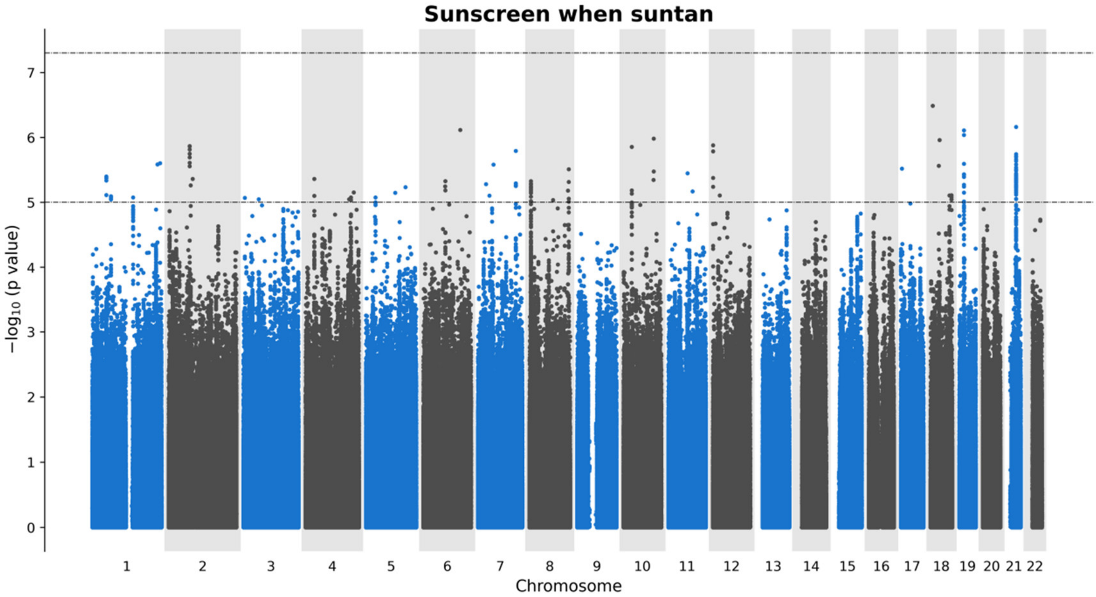
Sunscreen when suntan
−log10 (p value)
Chromosome
1 2 3 4 5 6 7 8 9 10 11 12 13 14 15 16 17 18 19 20 21 22

B) QQ Plots

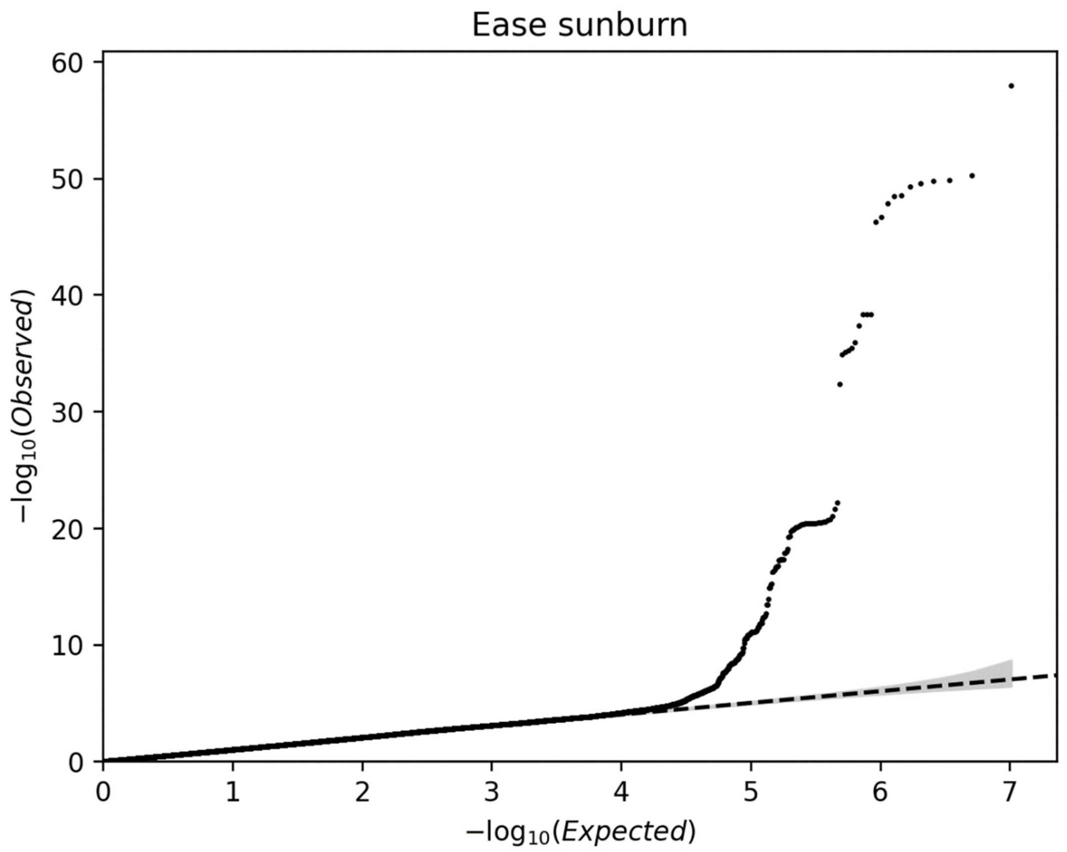

Ease sunburn
60
50
40
30
20
10
0
0
1
2
3
4
5
6
7
−log10(Observed)
−log10(Expected)

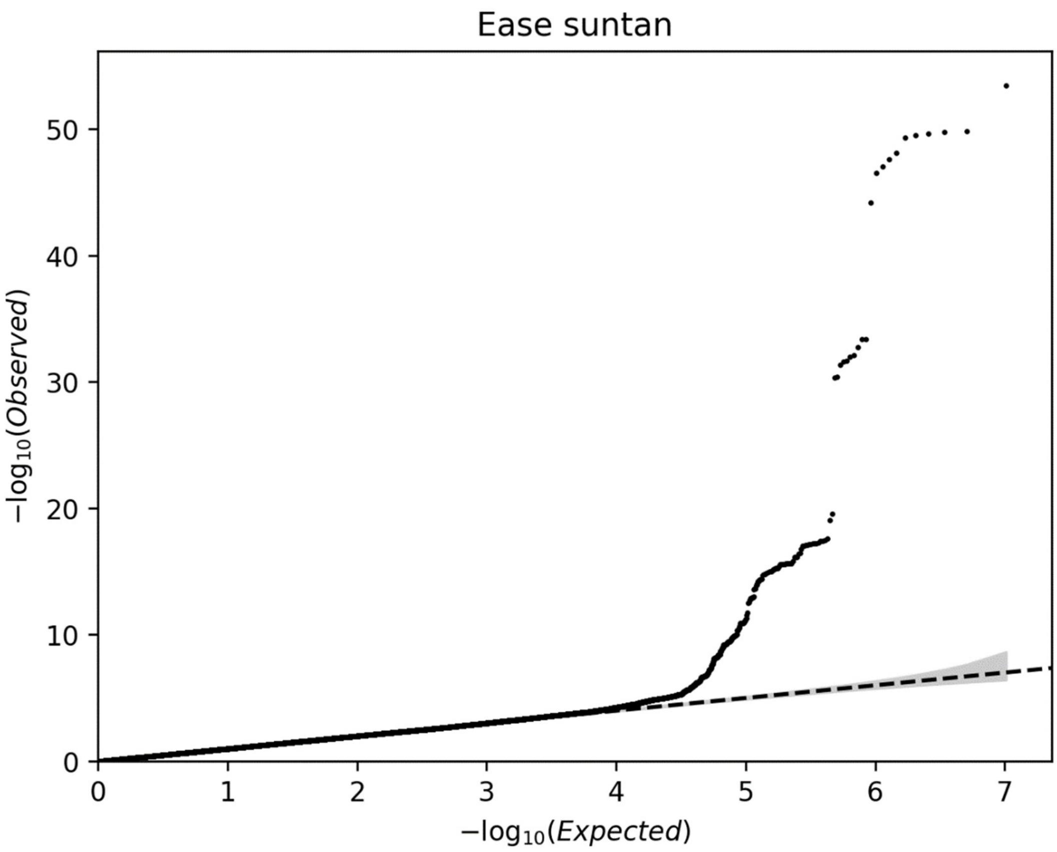

Ease suntan
0
10
20
30
40
50
$-\log_{10}(Observed)$
0
1
2
3
4
5
6
7
$-\log_{10}(Expected)$

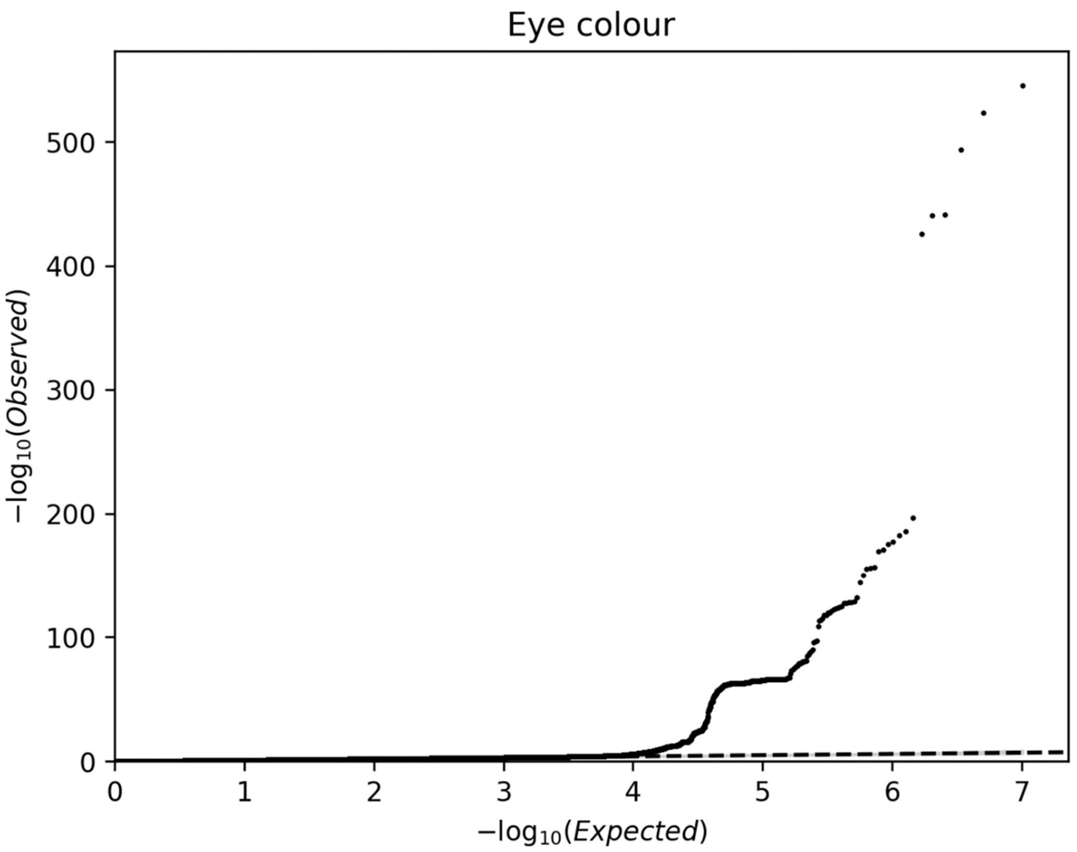

Eye colour
−log10(Observed)
−log10(Expected)
0
100
200
300
400
500
0
1
2
3
4
5
6
7

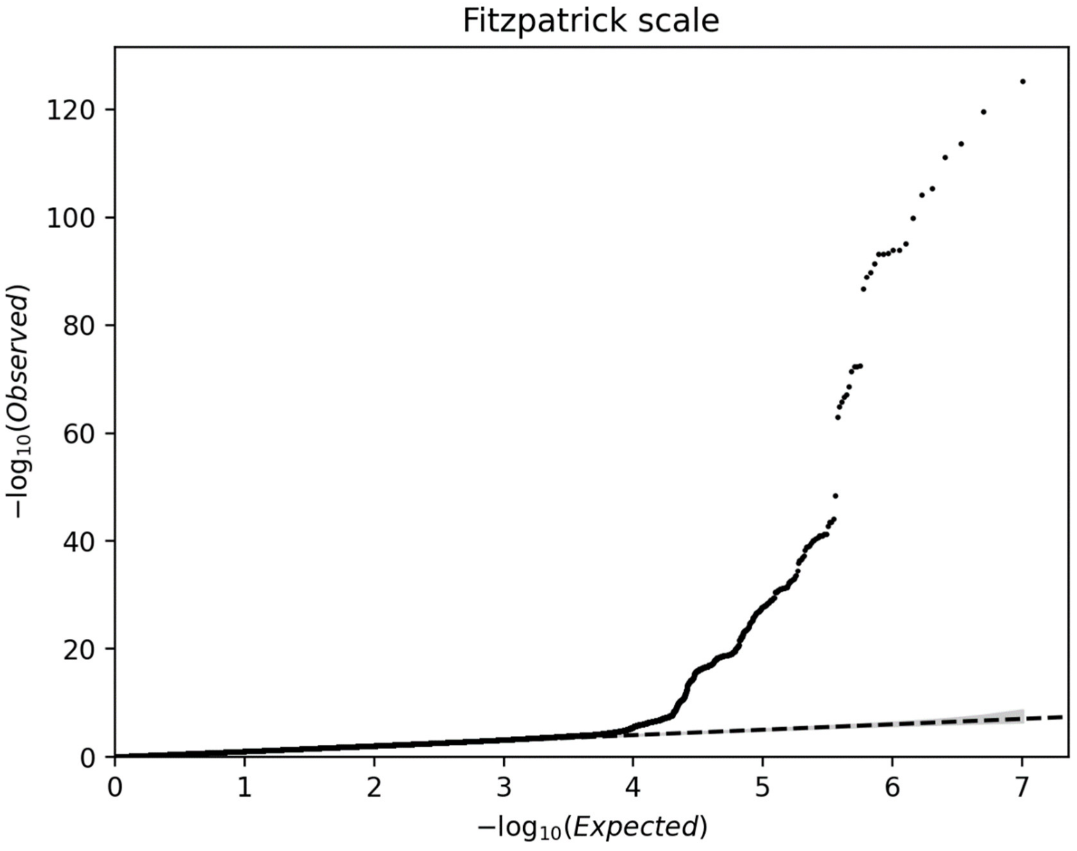

Fitzpatrick scale
−log10(Observed)
−log10(Expected)
0
20
40
60
80
100
120
0
1
2
3
4
5
6
7

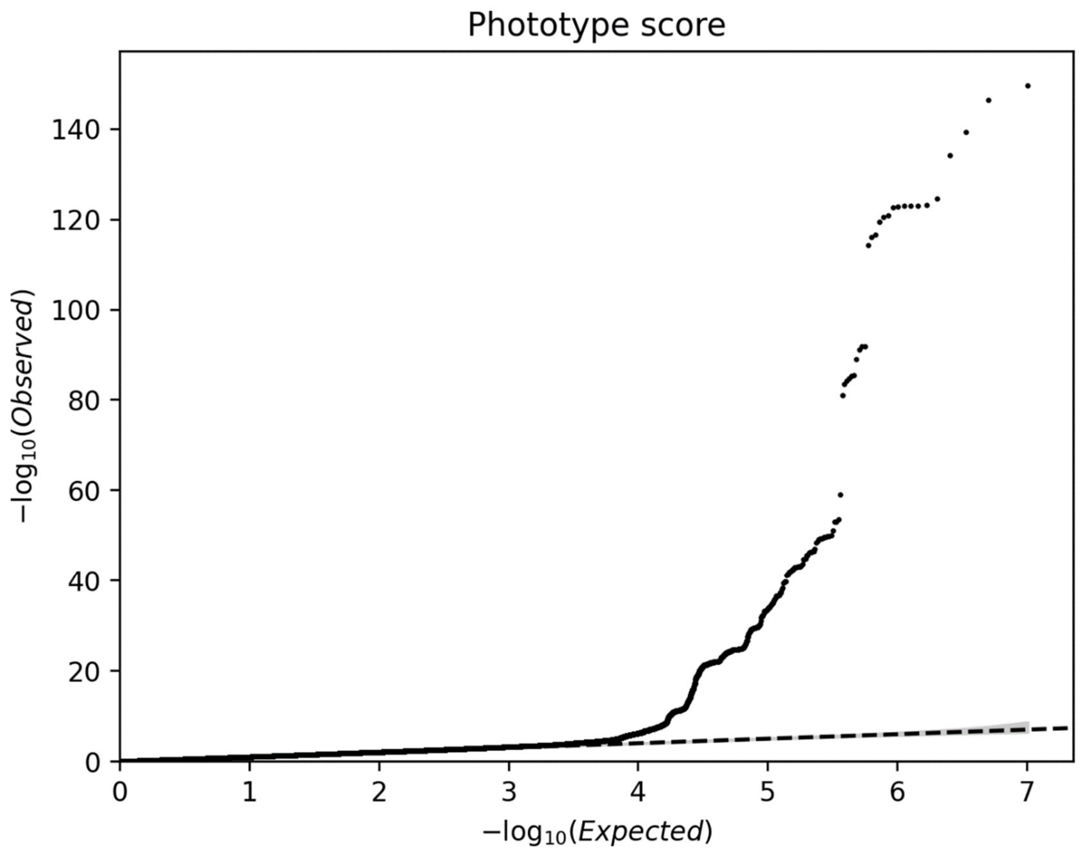
Phototype score
−log10(Observed)
−log10(Expected)
0
20
40
60
80
100
120
140
0
1
2
3
4
5
6
7

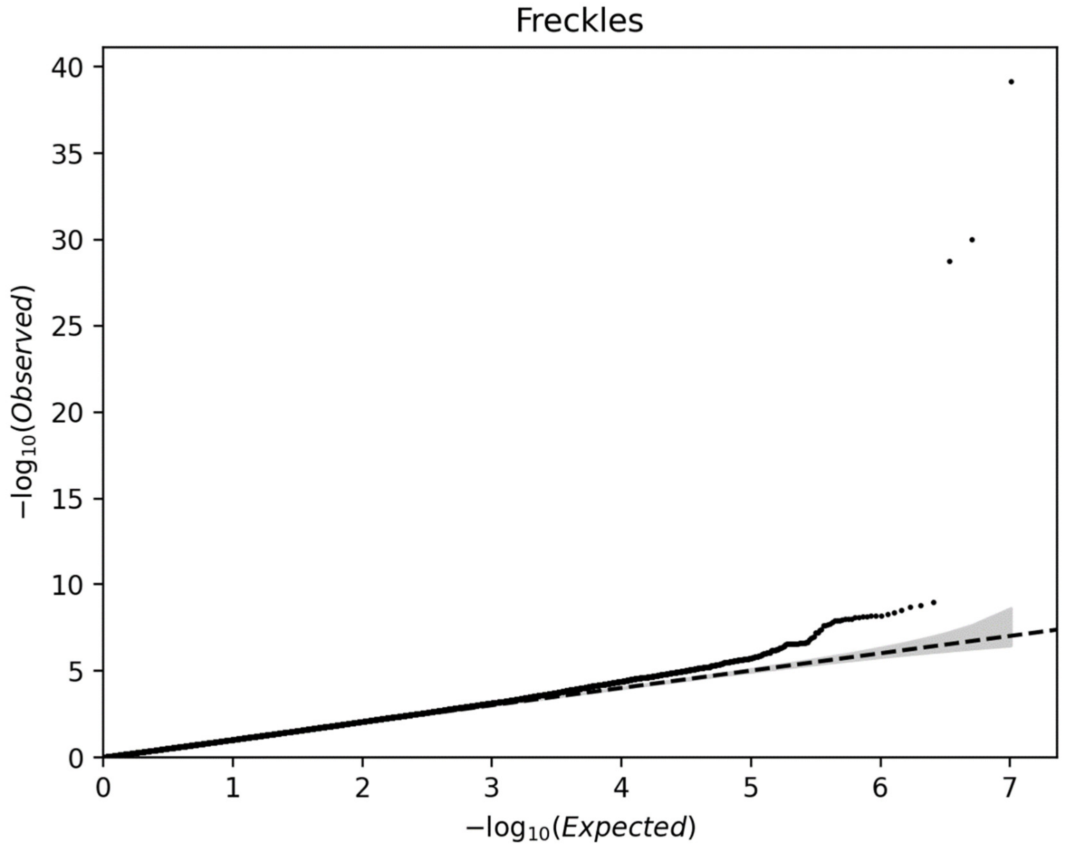
Freckles
−log10(Observed)
−log10(Expected)
0
5
10
15
20
25
30
35
40
0
1
2
3
4
5
6
7

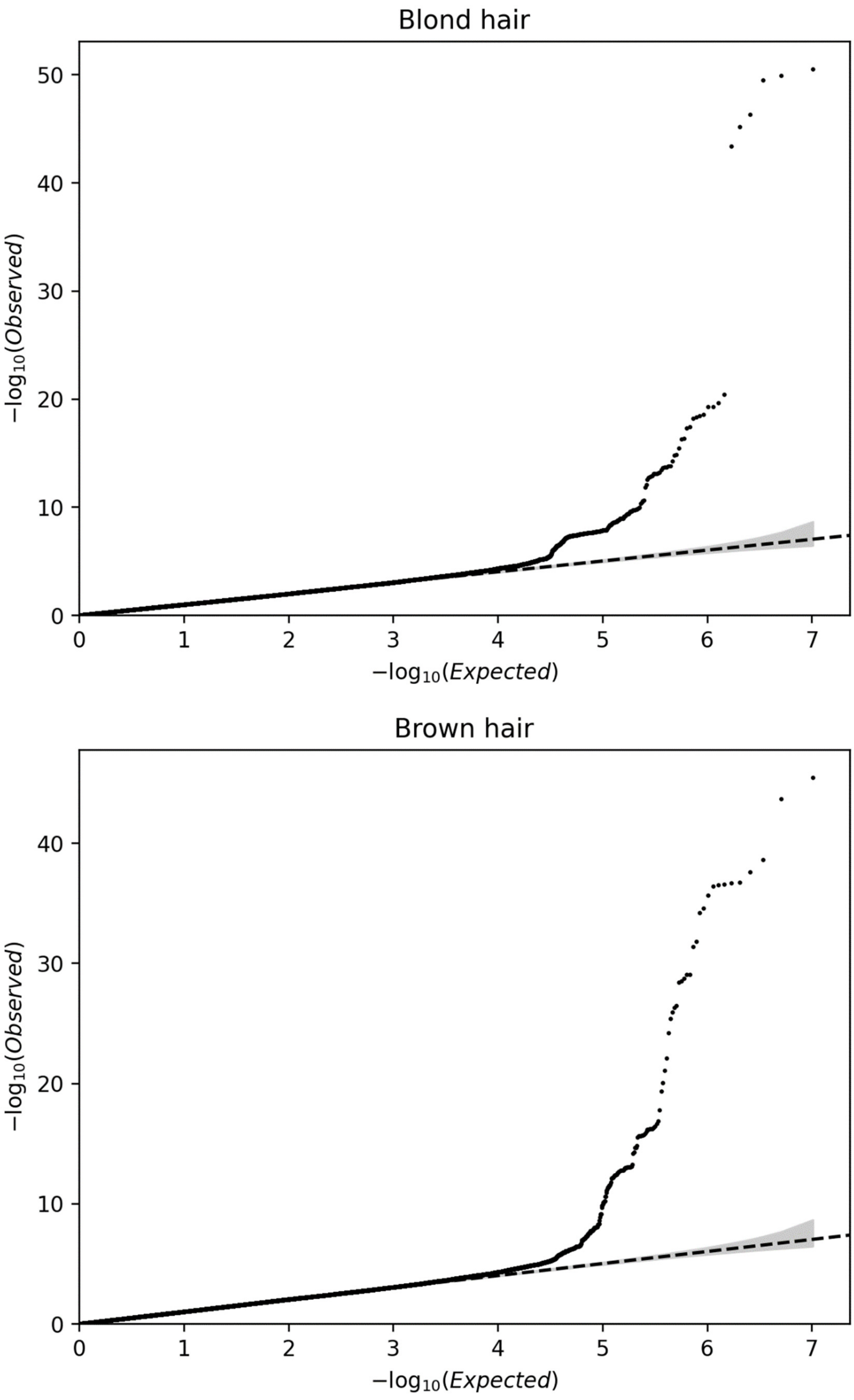

Blond hair
50
40
30
20
10
0
−log10(Observed)
0
1
2
3
4
5
6
7
−log10(Expected)
Brown hair
40
30
20
10
0
−log10(Observed)
0
1
2
3
4
5
6
7
−log10(Expected)

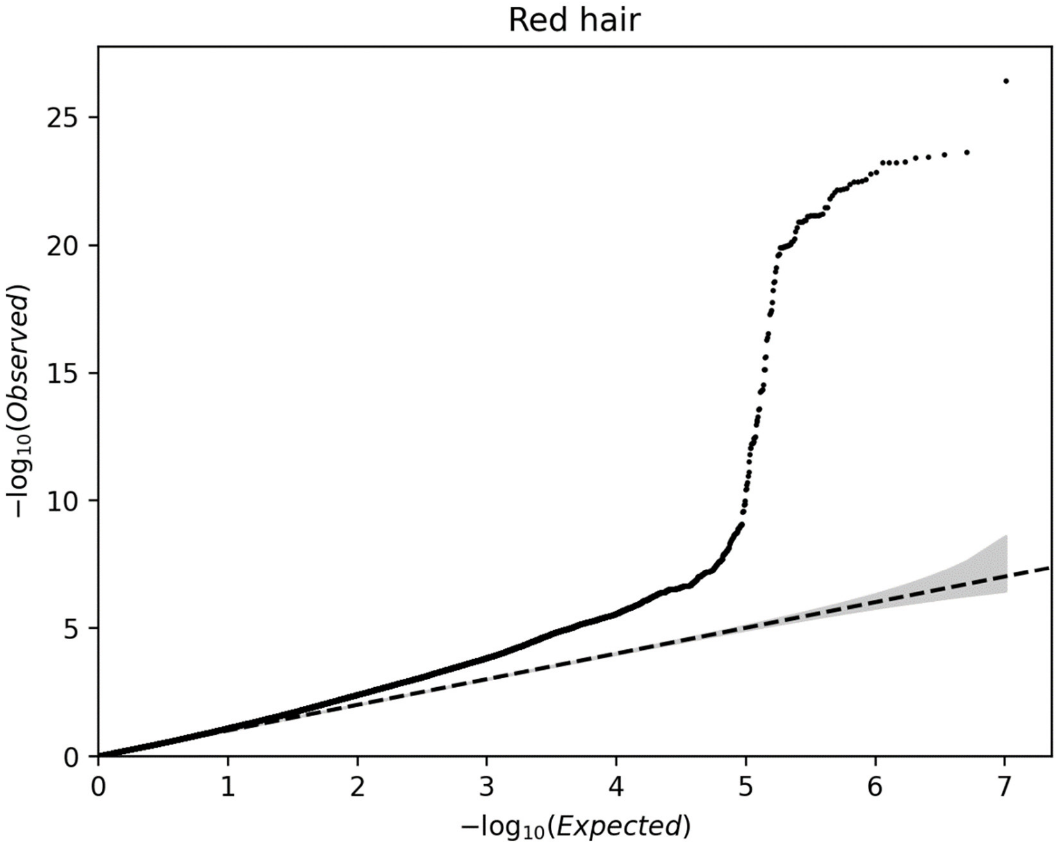
Red hair
−log10(Observed)
−log10(Expected)
0
5
10
15
20
25
0
1
2
3
4
5
6
7

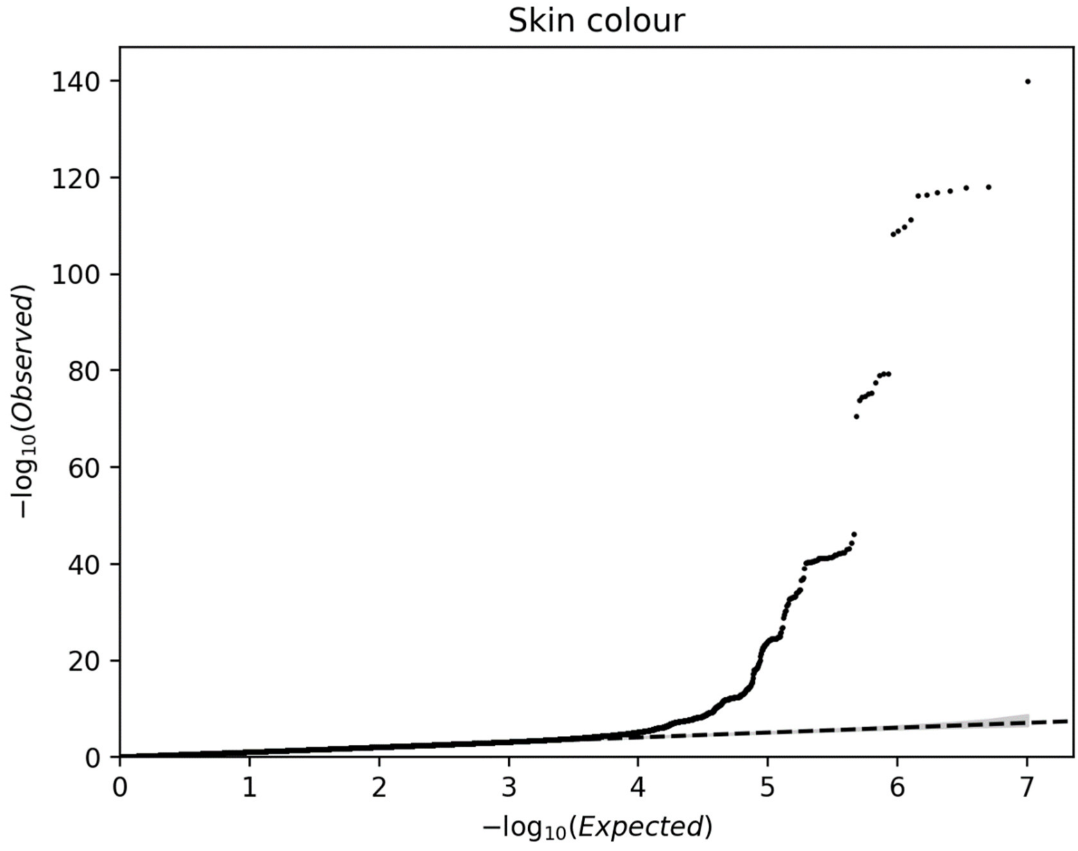
Skin colour
−log10(Observed)
−log10(Expected)
0
20
40
60
80
100
120
140
0
1
2
3
4
5
6
7

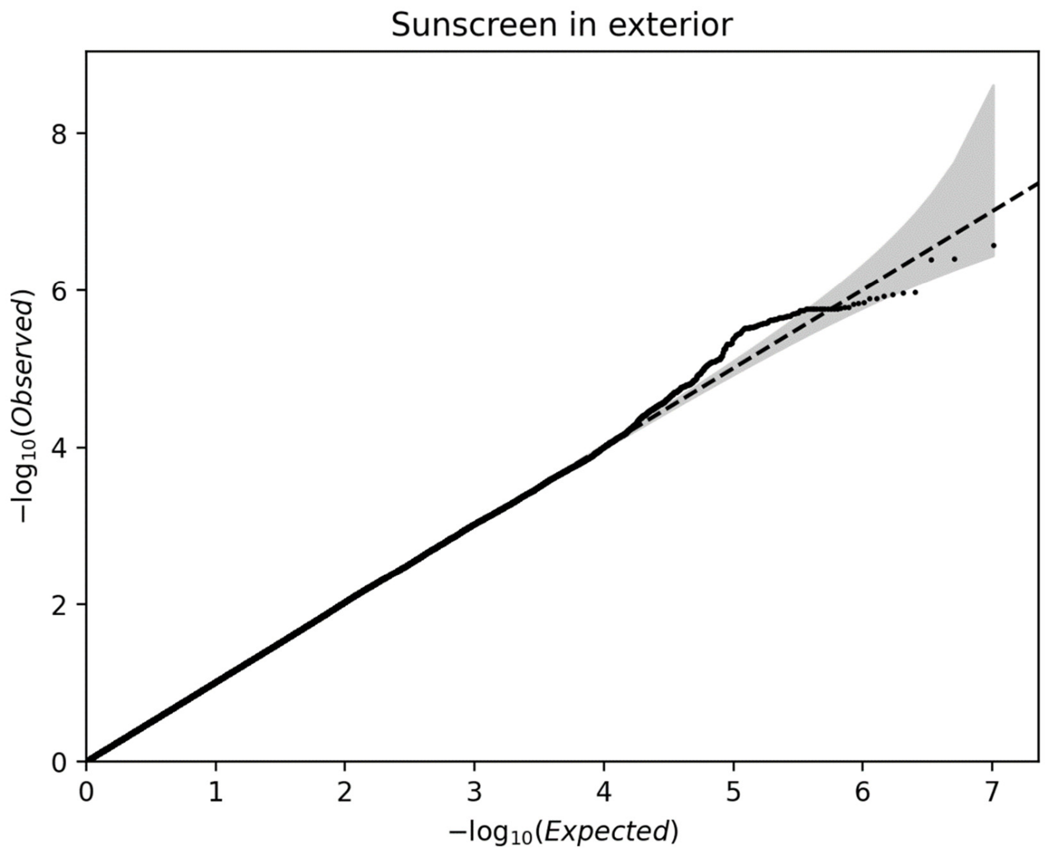

Sunscreen in exterior
−log10(Observed)
0
2
4
6
8
0
1
2
3
4
5
6
7
−log10(Expected)

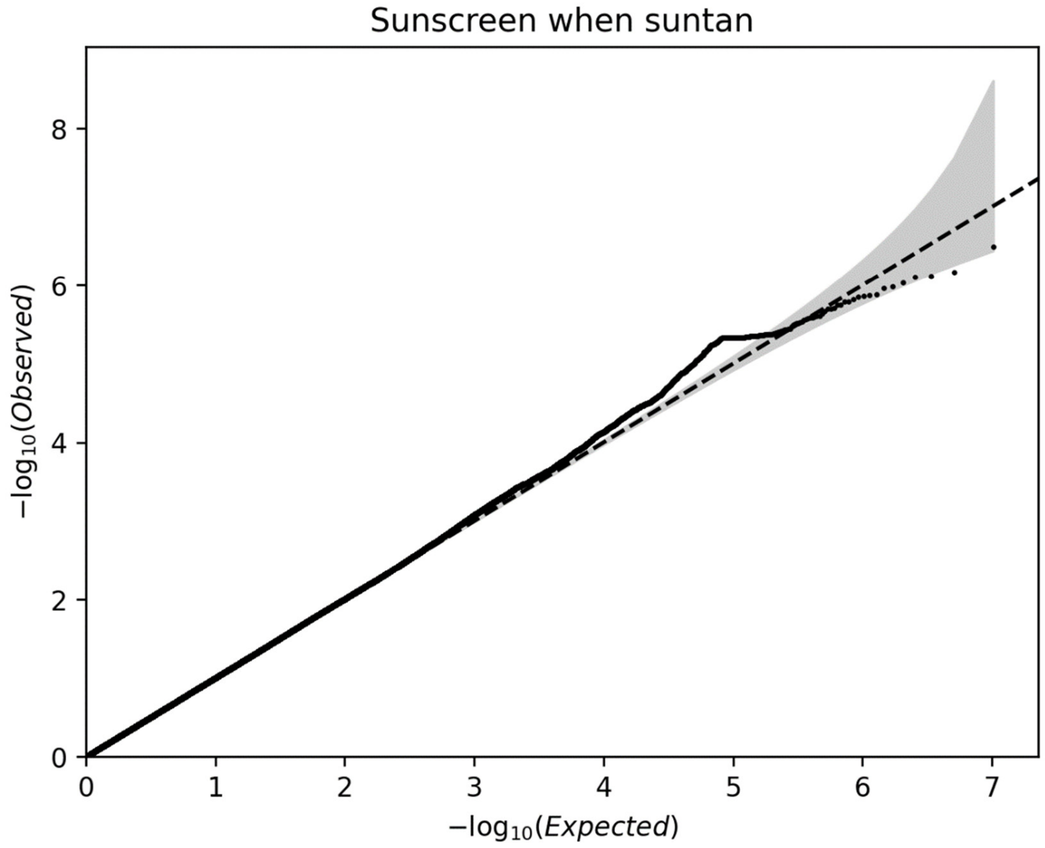

Sunscreen when suntan
−log10(Observed)
0
2
4
6
8
0
1
2
3
4
5
6
7
−log10(Expected)

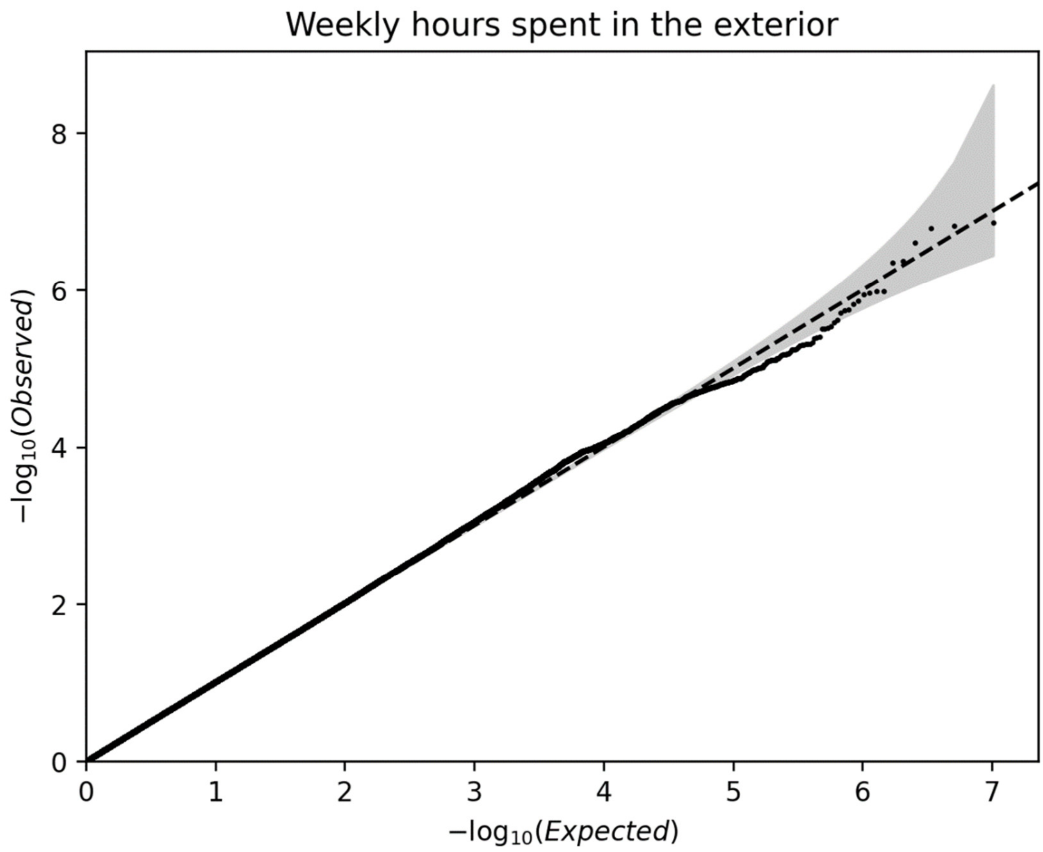

Weekly hours spent in the exterior
$-\log_{10}(Observed)$
$-\log_{10}(Expected)$
0
2
4
6
8
0
1
2
3
4
5
6
7

Supplementary Figure S4. Genetic Correlations among the 13 pigmentary traits. Results of the pair-wise genetic correlation of pigmentary traits expressed in r^{2}. Positive correlations are coloured in blue and negative correlations are coloured in red. Significant correlations are indicated with a white X. Significant positive genetic correlation was found between phototype score and Fitzpatrick scale, as expected because both are measures of the same phenotype. Significant negative genetic correlation was found between ease sunburn and both phototype traits, being fair phototype in higher risk of sunburn. Finally, significant negative genetic correlation was found between phototype score and brown hair, indicating that individuals with brown hair have lower phototypes than individuals with back hair.

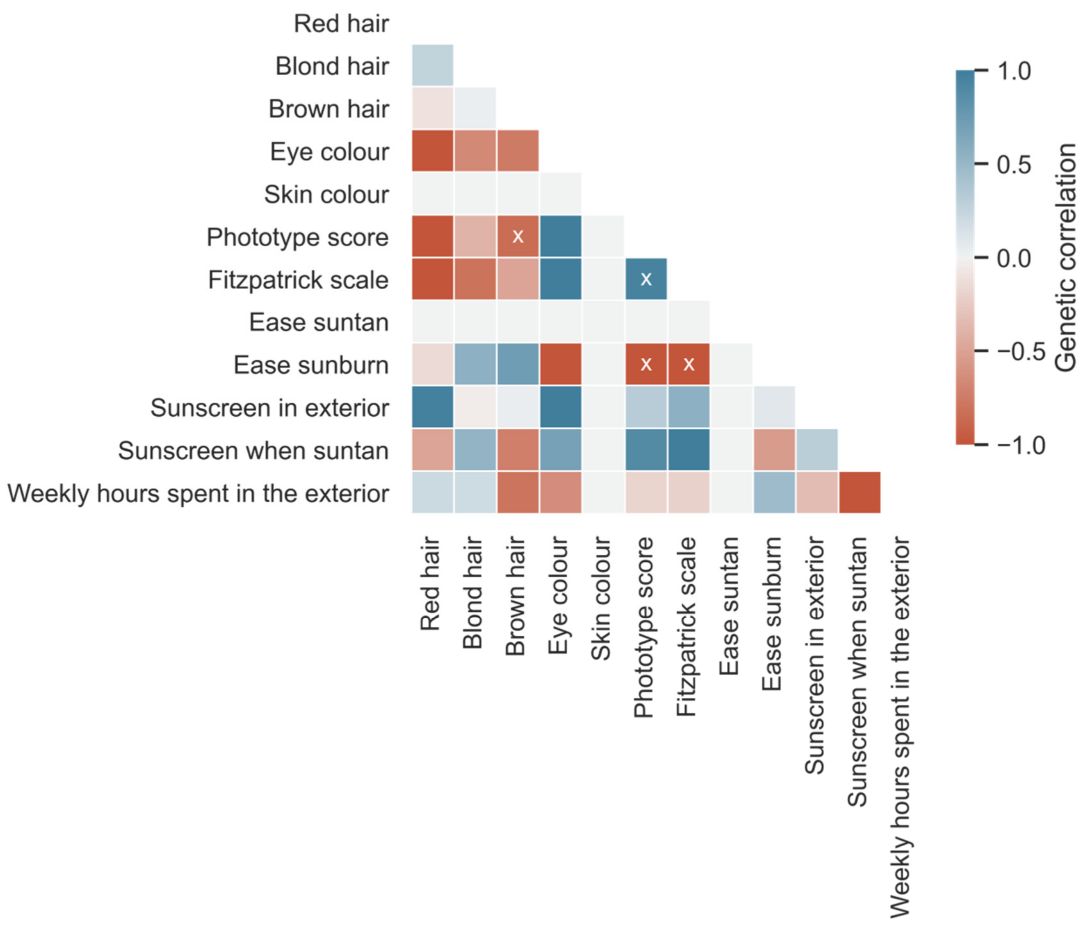

Supplementary Figure S5. Functional Enrichment in significant genes from gene-based analysis. Bar plots of the genes are showing in the X axis the enrichment ratio and in the Y axis the different results for the (A) GO terms, (B) pathways, (C) diseases and (D) phenotypes. Dark colour indicates that it passes the FDR threshold.

A) GO Terms

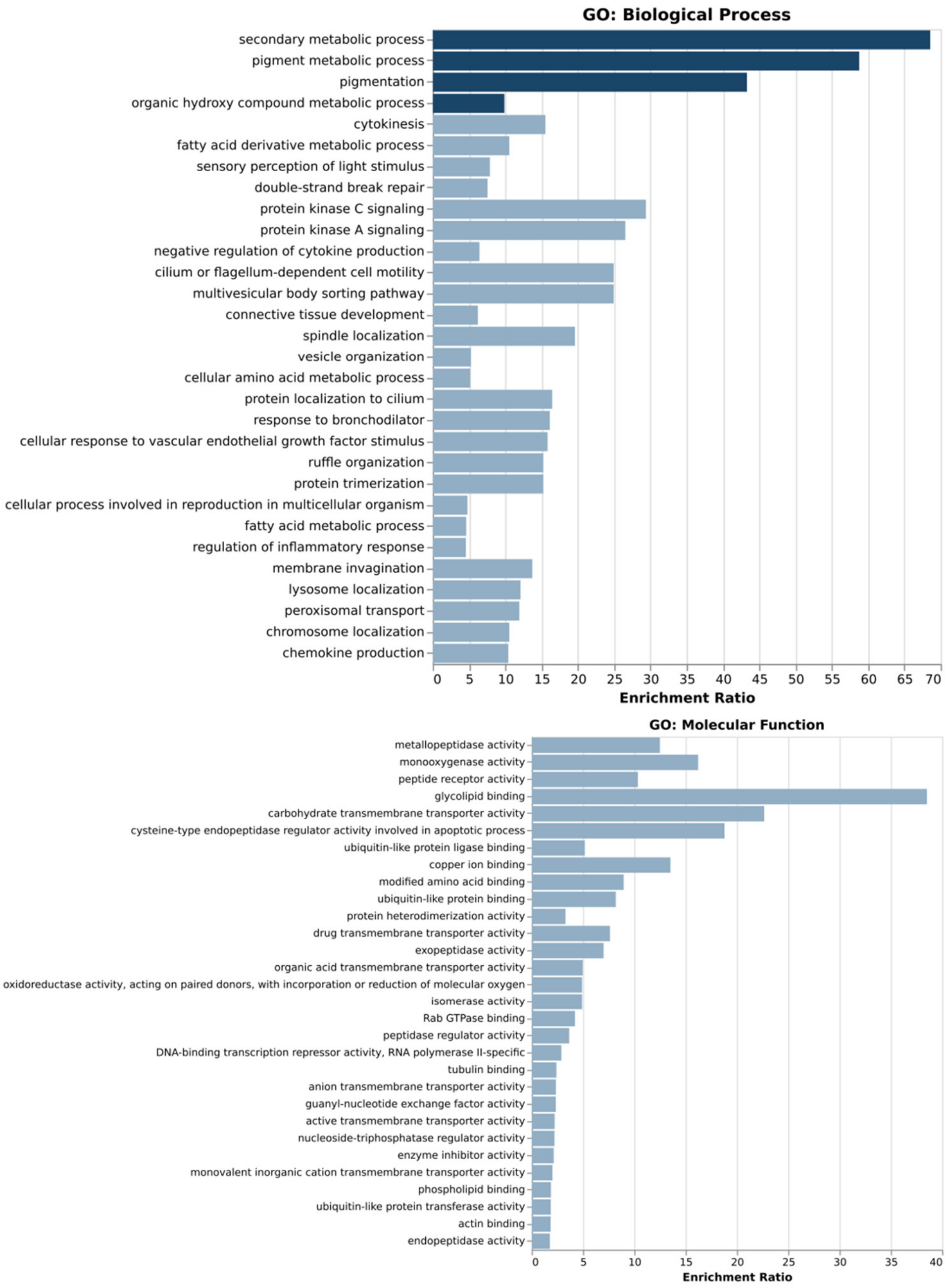

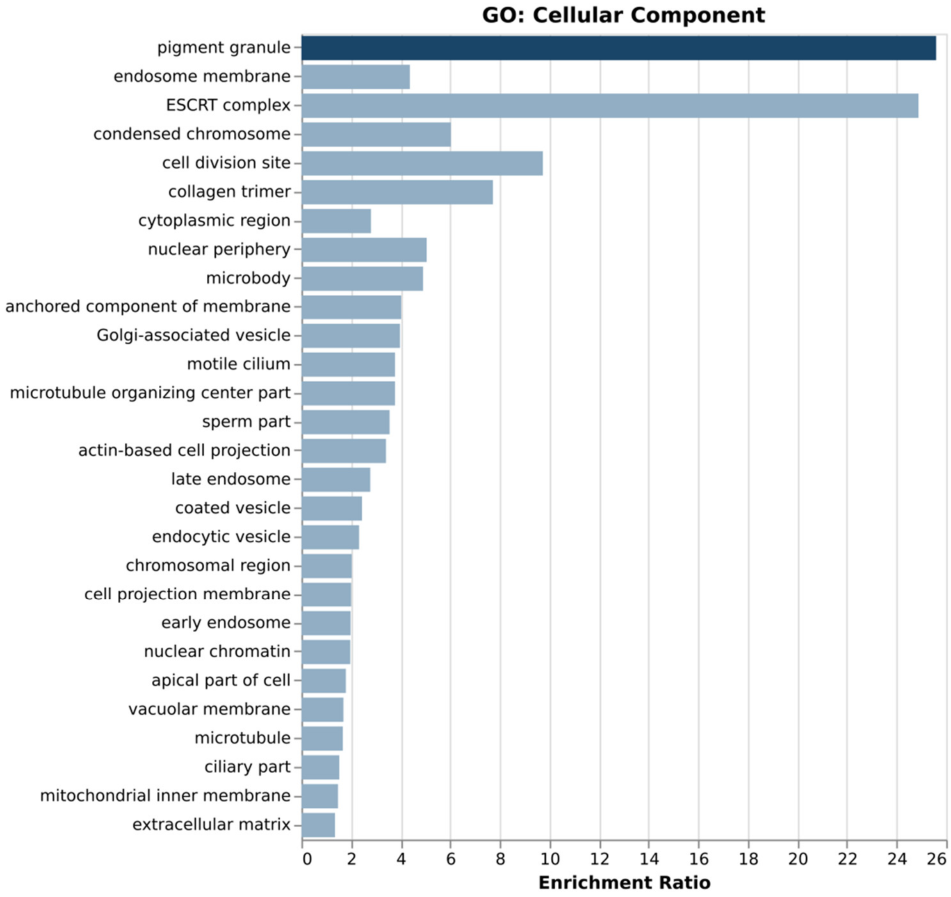

GO: Cellular Component
pigment granule
endosome membrane
ESCRT complex
condensed chromosome
cell division site
collagen trimer
cytoplasmic region
nuclear periphery
microbody
anchored component of membrane
Golgi-associated vesicle
motile cilium
microtubule organizing center part
sperm part
actin-based cell projection
late endosome
coated vesicle
endocytic vesicle
chromosomal region
cell projection membrane
early endosome
nuclear chromatin
apical part of cell
vacuolar membrane
microtubule
ciliary part
mitochondrial inner membrane
extracellular matrix
0 2 4 6 8 10 12 14 16 18 20 22 24 26
Enrichment Ratio

B) Pathways

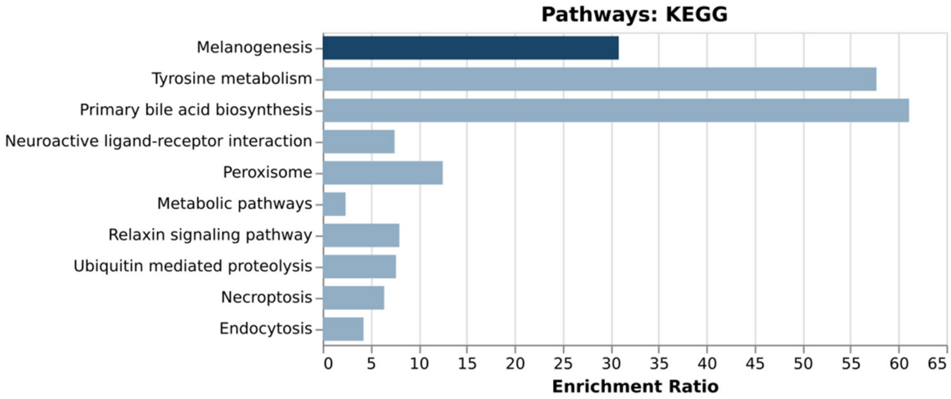

Pathways: KEGG
Melanogenesis
Tyrosine metabolism
Primary bile acid biosynthesis
Neuroactive ligand-receptor interaction
Peroxisome
Metabolic pathways
Relaxin signaling pathway
Ubiquitin mediated proteolysis
Necroptosis
Endocytosis
0 5 10 15 20 25 30 35 40 45 50 55 60 65
Enrichment Ratio

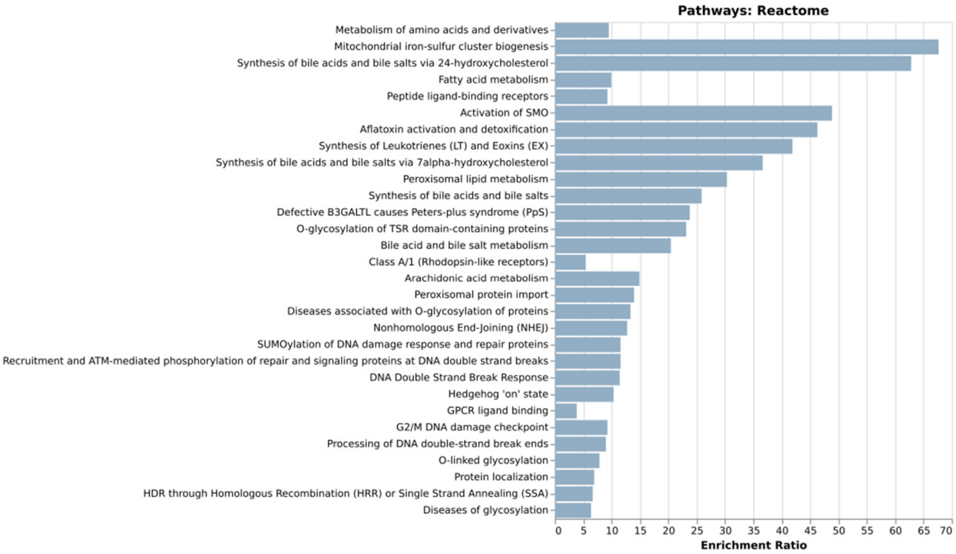
Pathways: Reactome
Metabolism of amino acids and derivatives
Mitochondrial iron-sulfur cluster biogenesis
Synthesis of bile acids and bile salts via 24-hydroxycholesterol
Fatty acid metabolism
Peptide ligand-binding receptors
Activation of SMO
Aflatoxin activation and detoxification
Synthesis of Leukotrienes (LT) and Eoxins (EX)
Synthesis of bile acids and bile salts via 7alpha-hydroxycholesterol
Peroxisomal lipid metabolism
Synthesis of bile acids and bile salts
Defective B3GALTL causes Peters-plus syndrome (PpS)
O-glycosylation of TSR domain-containing proteins
Bile acid and bile salt metabolism
Class A/1 (Rhodopsin-like receptors)
Arachidonic acid metabolism
Peroxisomal protein import
Diseases associated with O-glycosylation of proteins
Nonhomologous End-Joining (NHEJ)
SUMOylation of DNA damage response and repair proteins
Recruitment and ATM-mediated phosphorylation of repair and signaling proteins at DNA double strand breaks
DNA Double Strand Break Response
Hedgehog 'on' state
GPCR ligand binding
G2/M DNA damage checkpoint
Processing of DNA double-strand break ends
O-linked glycosylation
Protein localization
HDR through Homologous Recombination (HRR) or Single Strand Annealing (SSA)
Diseases of glycosylation
0 5 10 15 20 25 30 35 40 45 50 55 60 65 70
Enrichment Ratio

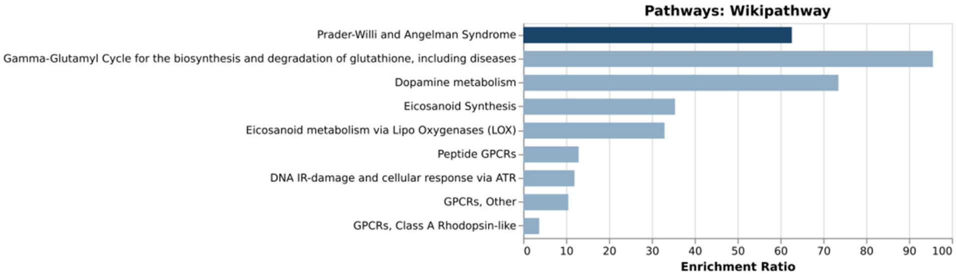
Pathways: Wikipathway
Prader-Willi and Angelman Syndrome
Gamma-Glutamyl Cycle for the biosynthesis and degradation of glutathione, including diseases
Dopamine metabolism
Eicosanoid Synthesis
Eicosanoid metabolism via Lipo Oxygenases (LOX)
Peptide GPCRs
DNA IR-damage and cellular response via ATR
GPCRs, Other
GPCRs, Class A Rhodopsin-like
0 10 20 30 40 50 60 70 80 90 100
Enrichment Ratio

C) Diseases

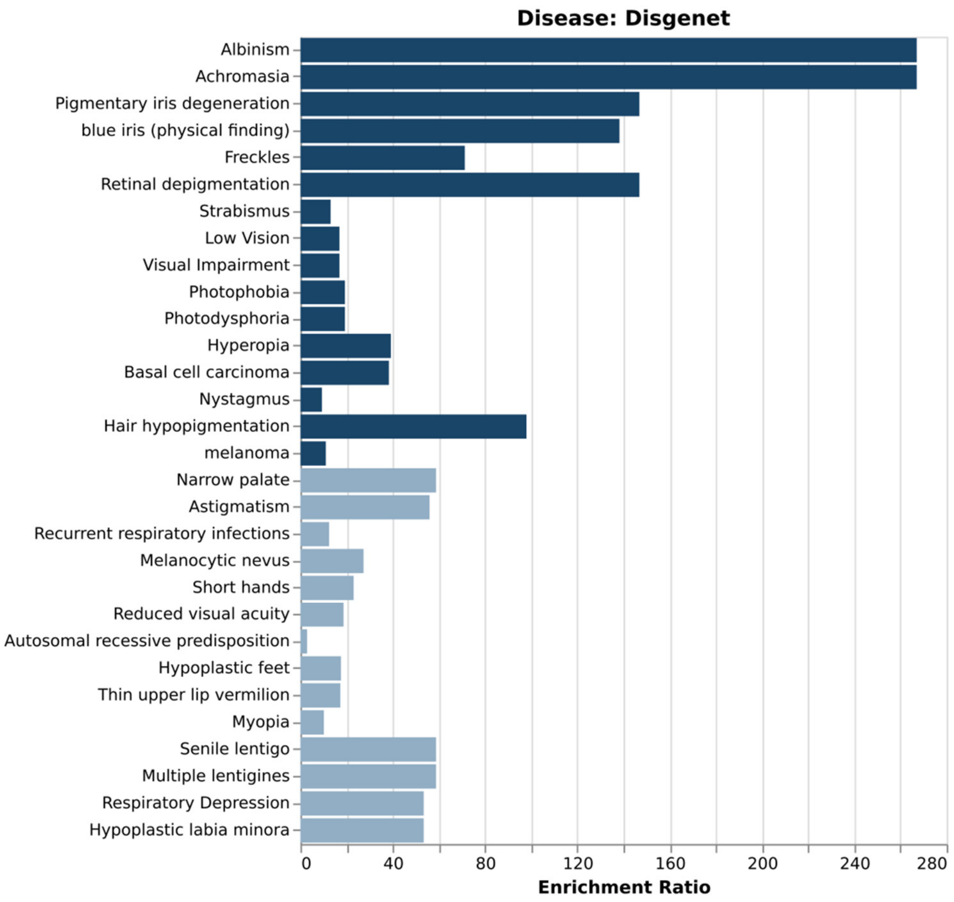

Disease: Disgenet
Albinism
Achromasia
Pigmentary iris degeneration
blue iris (physical finding)
Freckles
Retinal depigmentation
Strabismus
Low Vision
Visual Impairment
Photophobia
Photodysphoria
Hyperopia
Basal cell carcinoma
Nystagmus
Hair hypopigmentation
melanoma
Narrow palate
Astigmatism
Recurrent respiratory infections
Melanocytic nevus
Short hands
Reduced visual acuity
Autosomal recessive predisposition
Hypoplastic feet
Thin upper lip vermilion
Myopia
Senile lentigo
Multiple lentigines
Respiratory Depression
Hypoplastic labia minora
0
40
80
120
160
200
240
280
Enrichment Ratio

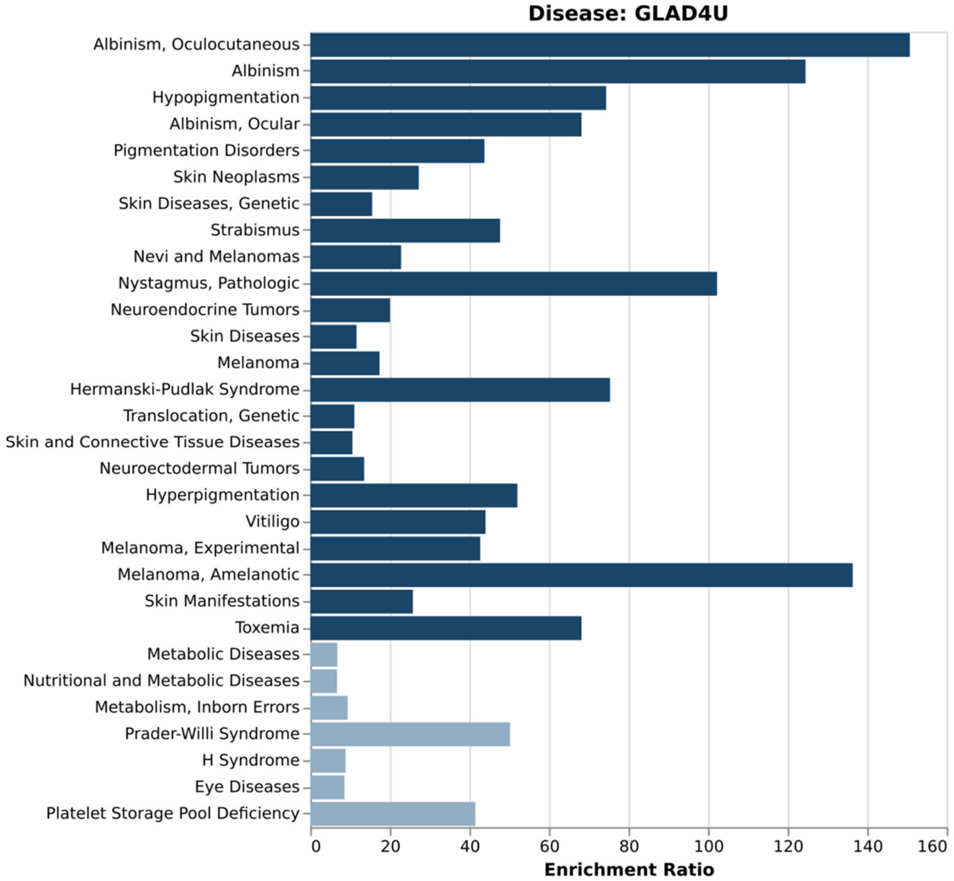

Disease: GLAD4U
Albinism, Oculocutaneous
Albinism
Hypopigmentation
Albinism, Ocular
Pigmentation Disorders
Skin Neoplasms
Skin Diseases, Genetic
Strabismus
Nevi and Melanomas
Nystagmus, Pathologic
Neuroendocrine Tumors
Skin Diseases
Melanoma
Hermanski-Pudlak Syndrome
Translocation, Genetic
Skin and Connective Tissue Diseases
Neuroectodermal Tumors
Hyperpigmentation
Vitiligo
Melanoma, Experimental
Melanoma, Amelanotic
Skin Manifestations
Toxemia
Metabolic Diseases
Nutritional and Metabolic Diseases
Metabolism, Inborn Errors
Prader-Willi Syndrome
H Syndrome
Eye Diseases
Platelet Storage Pool Deficiency
0
20
40
60
80
100
120
140
160
Enrichment Ratio

D) Phenotypes

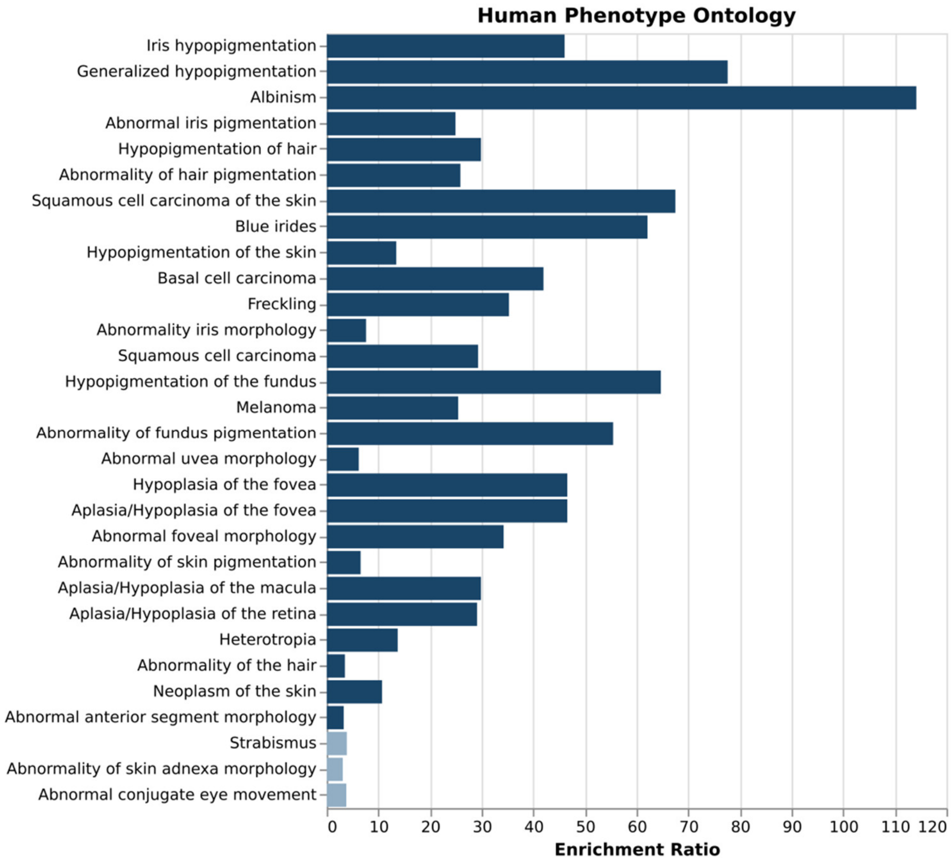

Supplementary Figure S6. LocusZoom of genome-wide association analysis of structural variants (n = 15) and trait combination reaching the suggestive threshold (n = 38). Plots show the –log10 p-values (Y axis) indicating the base pair (BP) location of the corresponding chromosome (X axis) together with mapping genes. Blue horizontal dashed line represents the 1e-6 threshold and red horizonal dashed line represents the genome-wide significant (5e-8). In purple is represented the SV and the other colours indicate the LD with it.

1:212027531:T<midDEL>

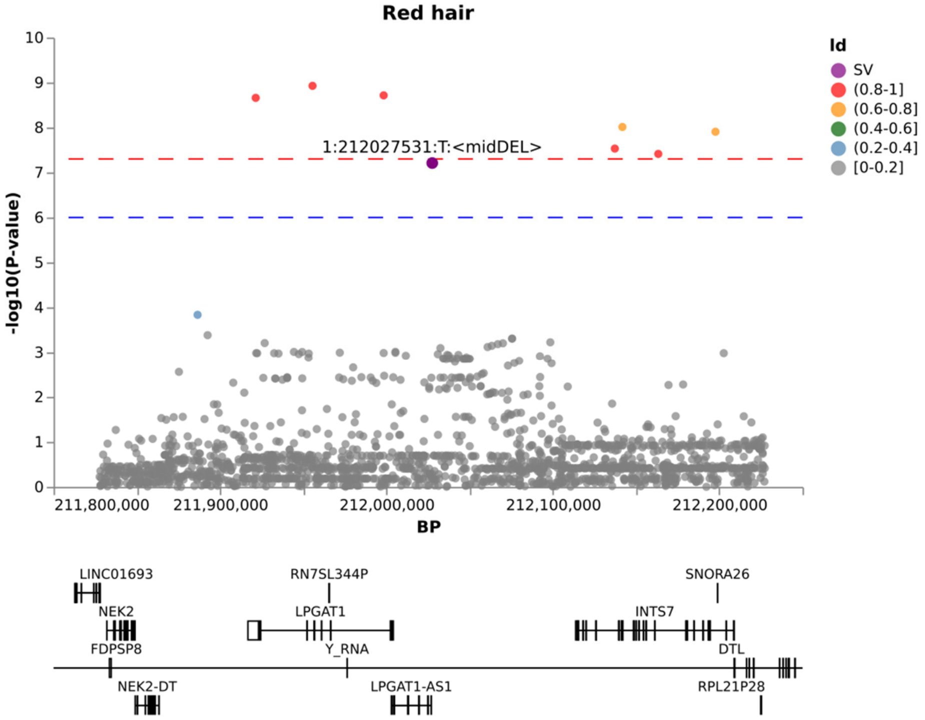

5:33973937:G:<midDEL>

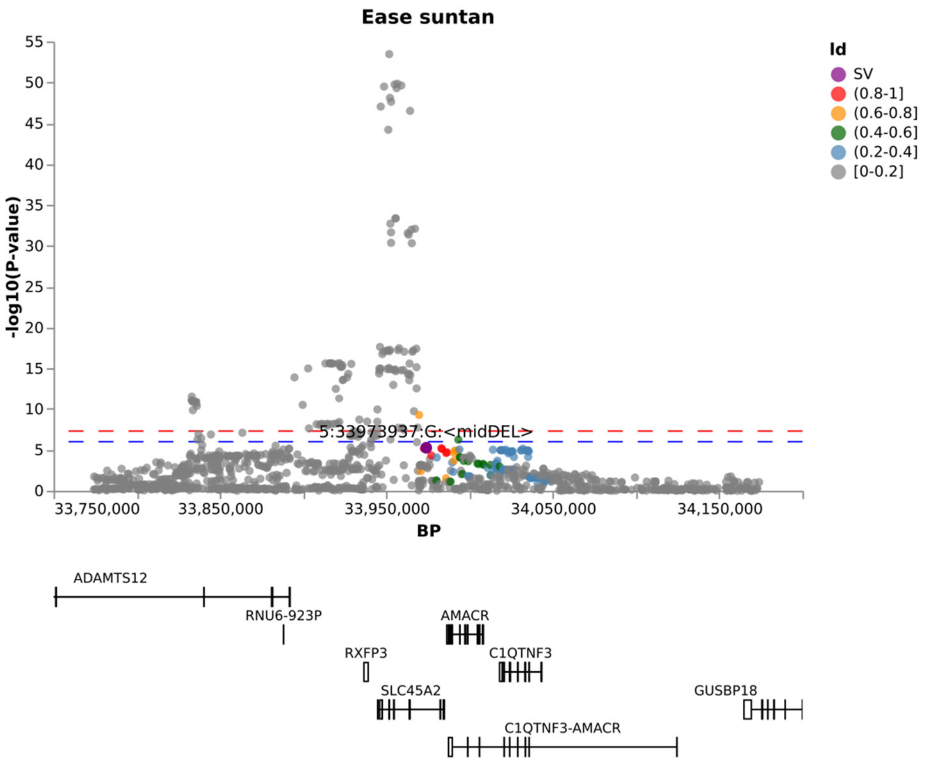

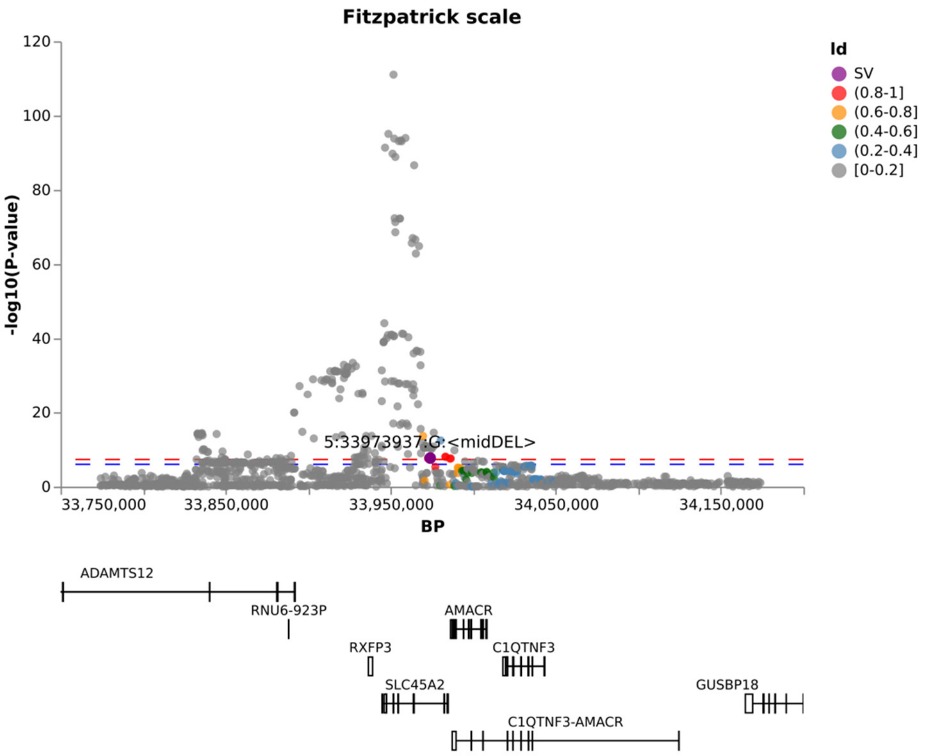

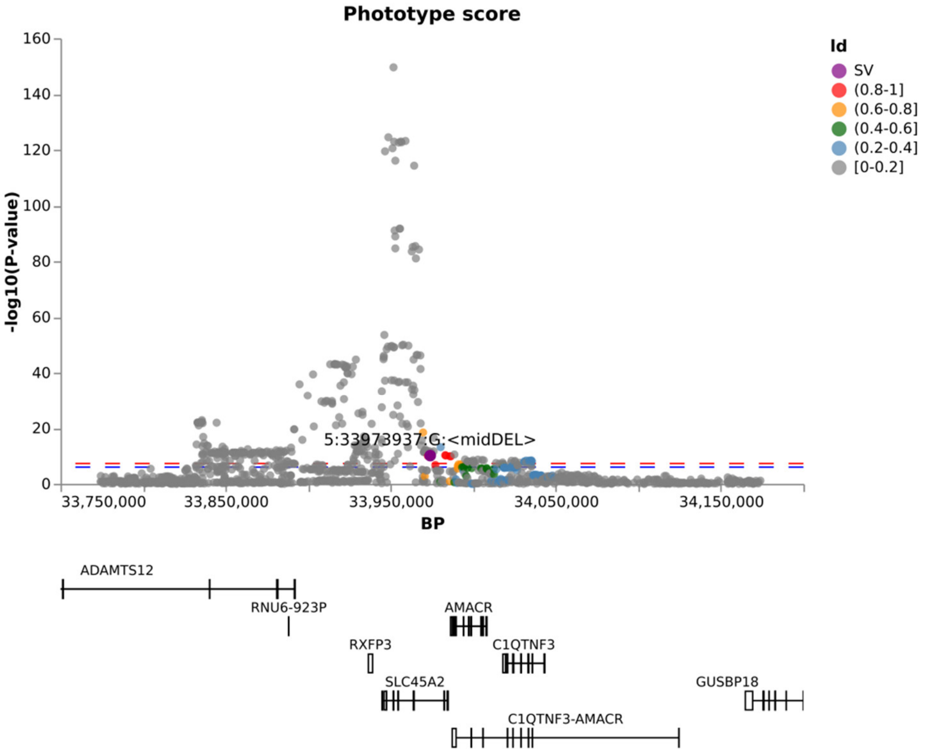

Phototype score
160
140
120
100
80
60
40
20
0
-log10(P-value)
5:33973937:G:<midDEL>
33,750,000
33,850,000
33,950,000
34,050,000
34,150,000
BP
ld
SV
(0.8-1]
(0.6-0.8]
(0.4-0.6]
(0.2-0.4]
[0-0.2]
ADAMTS12
RNU6-923P
AMACR
RXFP3
C1QTNF3
SLC45A2
GUSBP18
C1QTNF3-AMACR

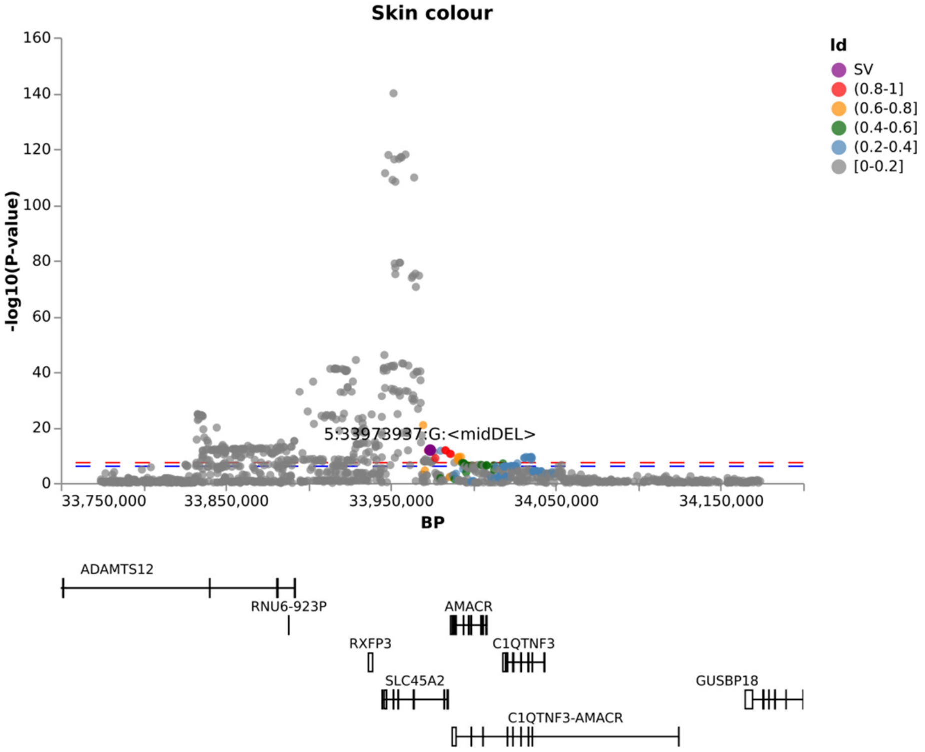

Skin colour
160
140
120
100
80
60
40
20
0
-log10(P-value)
5:33973937:G:<midDEL>
33,750,000
33,850,000
33,950,000
34,050,000
34,150,000
BP
ld
SV
(0.8-1]
(0.6-0.8]
(0.4-0.6]
(0.2-0.4]
[0-0.2]
ADAMTS12
RNU6-923P
AMACR
RXFP3
C1QTNF3
SLC45A2
GUSBP18
C1QTNF3-AMACR

6:56387576:C:<INS:ME:ALU>

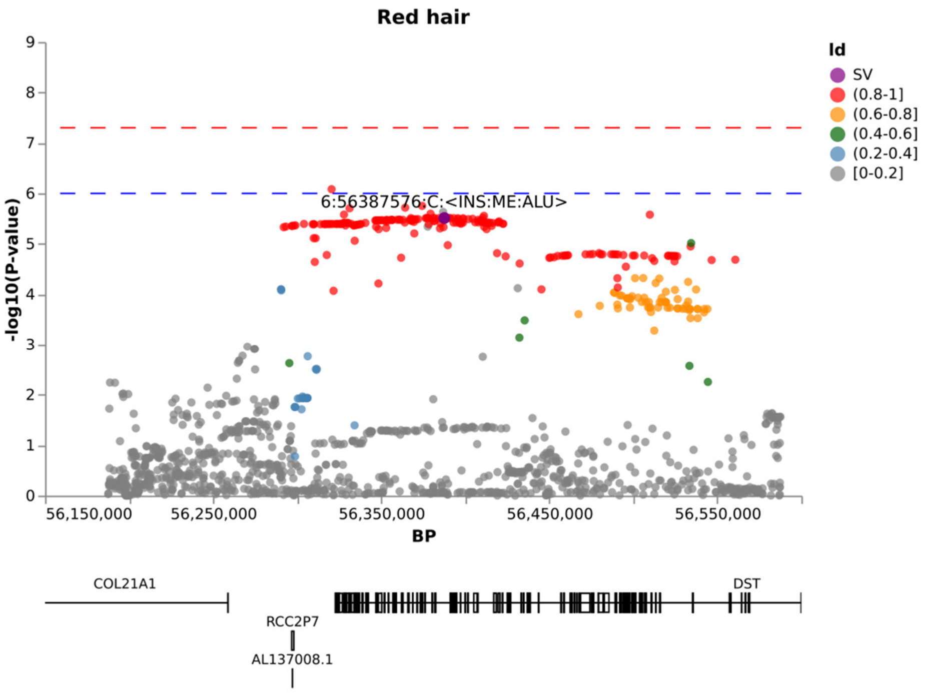

7:64921798:G:<TRA>

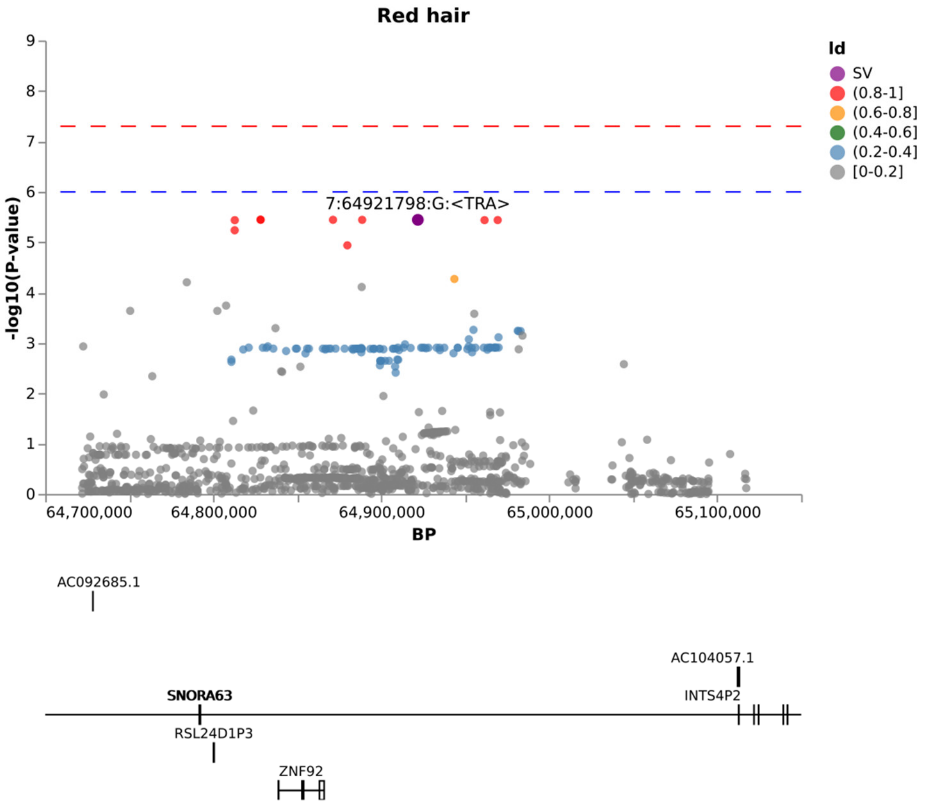

9:12756780:C:<midDEL>

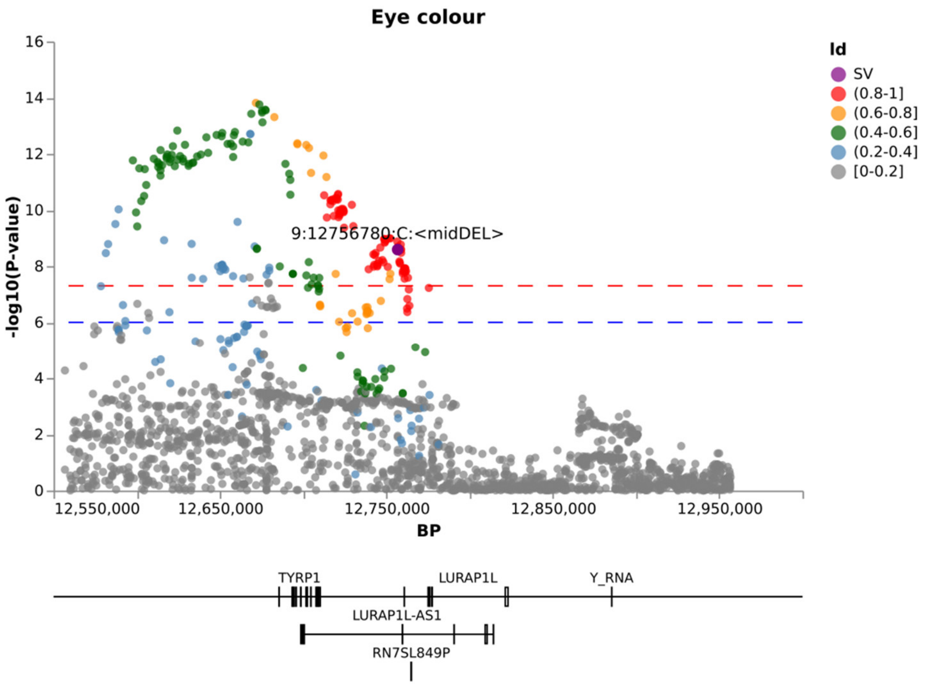

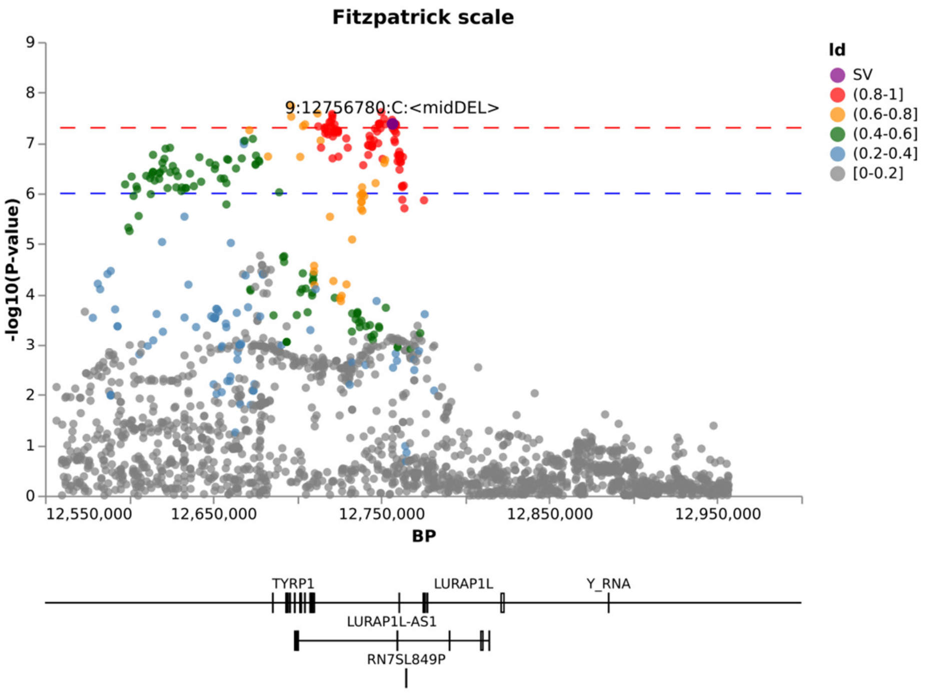
Fitzpatrick scale
-log10(P-value)
9:12756780:C:<midDEL>
BP
12,550,000
12,650,000
12,750,000
12,850,000
12,950,000
ld
SV
(0.8-1]
(0.6-0.8]
(0.4-0.6]
(0.2-0.4]
[0-0.2]
TYRP1
LURAP1L
Y_RNA
LURAP1L-AS1
RN7SL849P

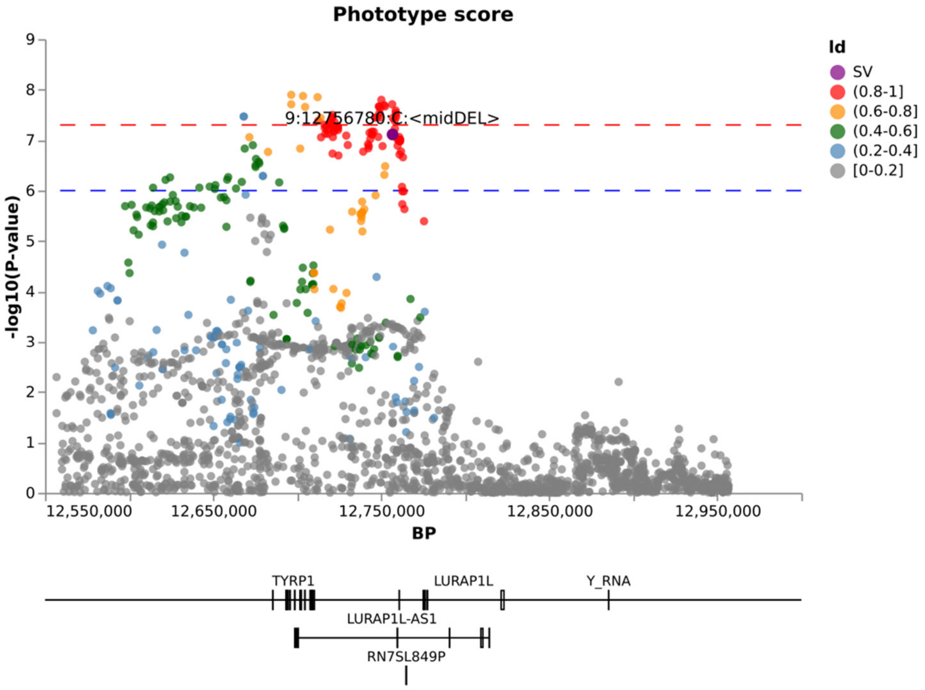
Phototype score
-log10(P-value)
9:12756780:C:<midDEL>
BP
12,550,000
12,650,000
12,750,000
12,850,000
12,950,000
ld
SV
(0.8-1]
(0.6-0.8]
(0.4-0.6]
(0.2-0.4]
[0-0.2]
TYRP1
LURAP1L
Y_RNA
LURAP1L-AS1
RN7SL849P

9:16876769:G:<midDEL>

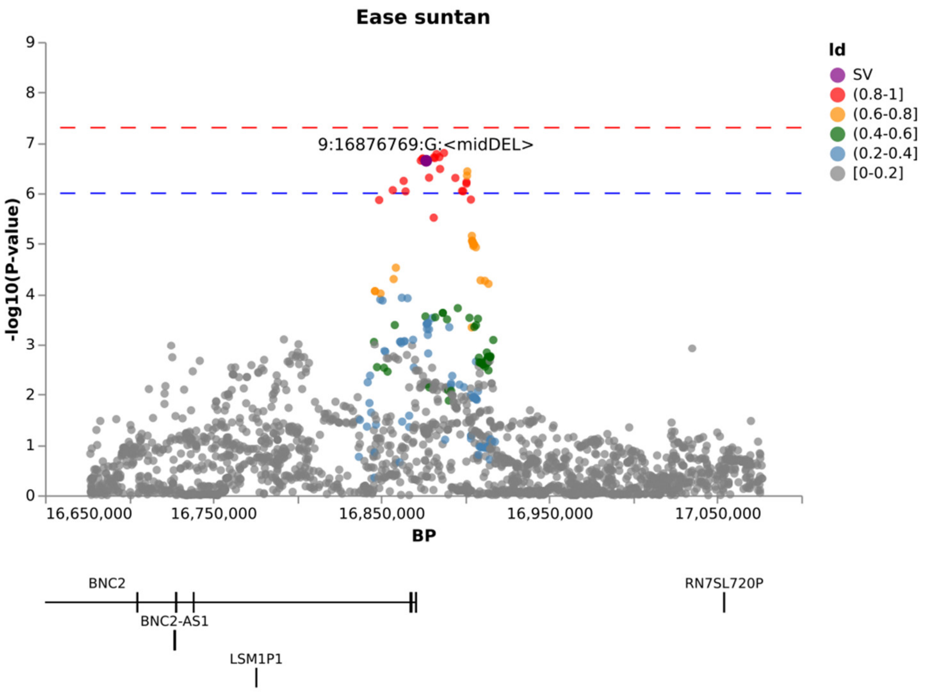

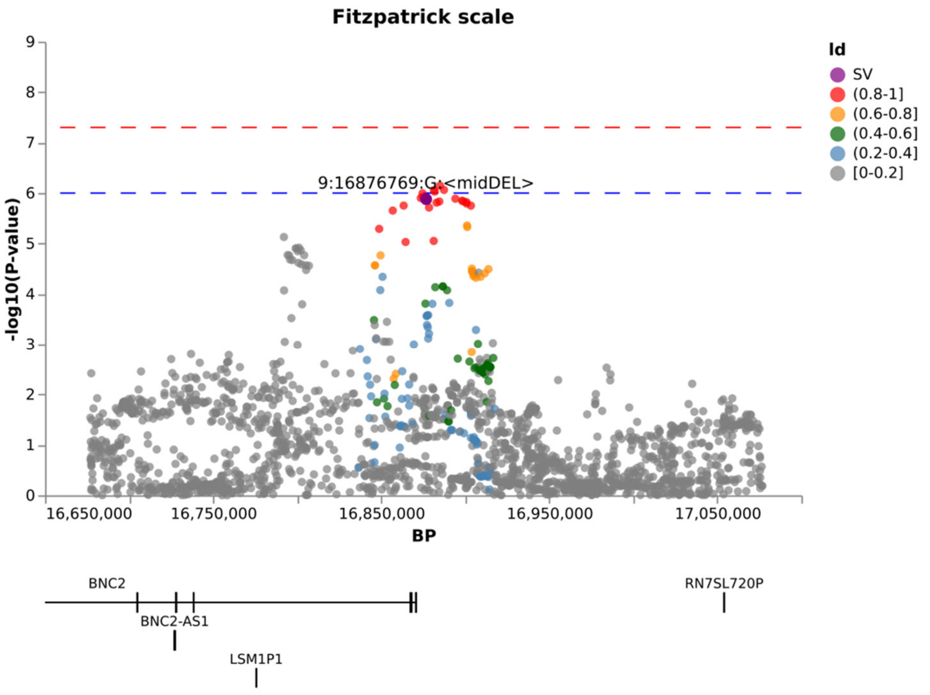

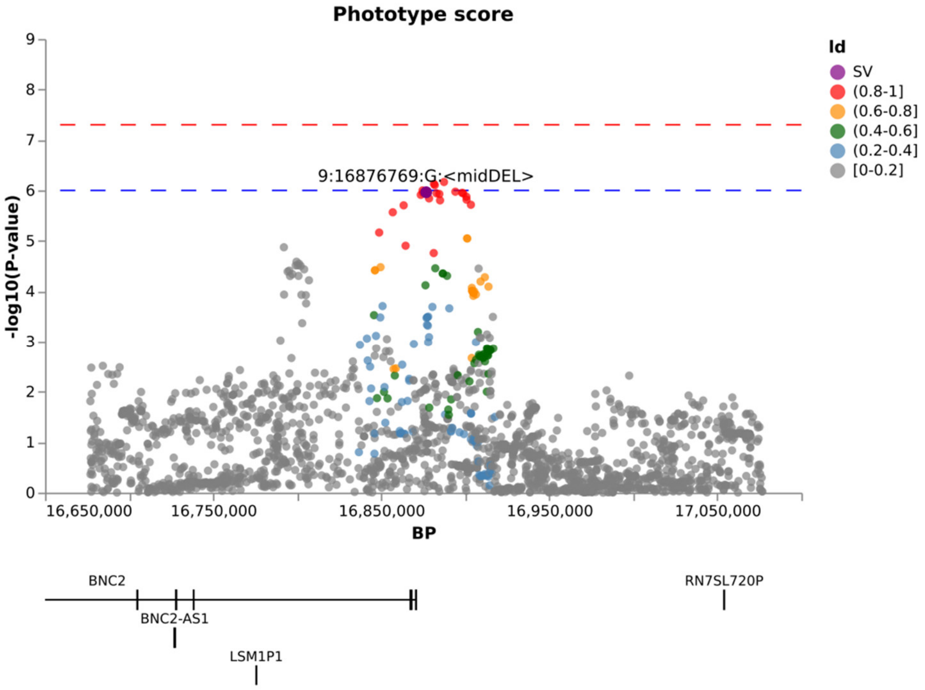
Phototype score
9:16876769:G:<midDEL>
-log10(P-value)
BP
16,650,000
16,750,000
16,850,000
16,950,000
17,050,000
ld
SV
(0.8-1]
(0.6-0.8]
(0.4-0.6]
(0.2-0.4]
[0-0.2]
BNC2
BNC2-AS1
LSM1P1
RN7SL720P

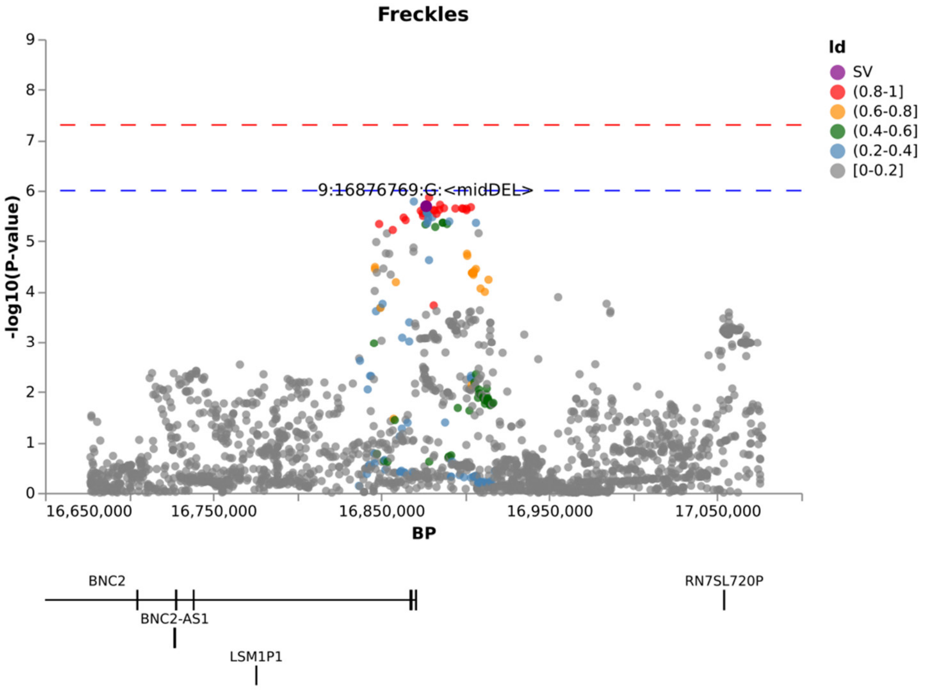
Freckles
9:16876769:G:<midDEL>
-log10(P-value)
BP
16,650,000
16,750,000
16,850,000
16,950,000
17,050,000
ld
SV
(0.8-1]
(0.6-0.8]
(0.4-0.6]
(0.2-0.4]
[0-0.2]
BNC2
BNC2-AS1
LSM1P1
RN7SL720P

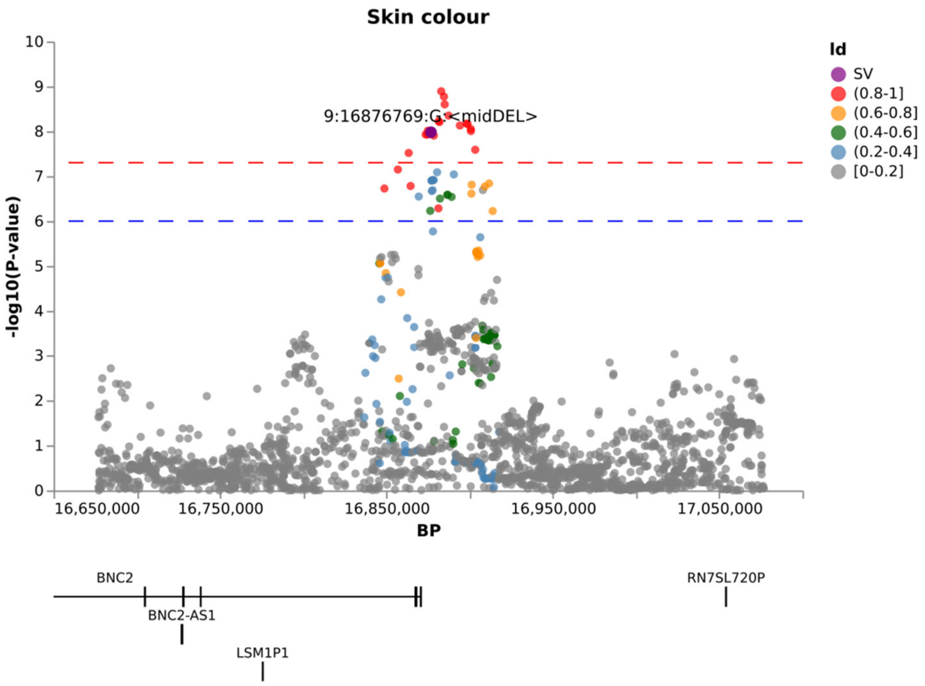

20:134809221:A:<midDEL>

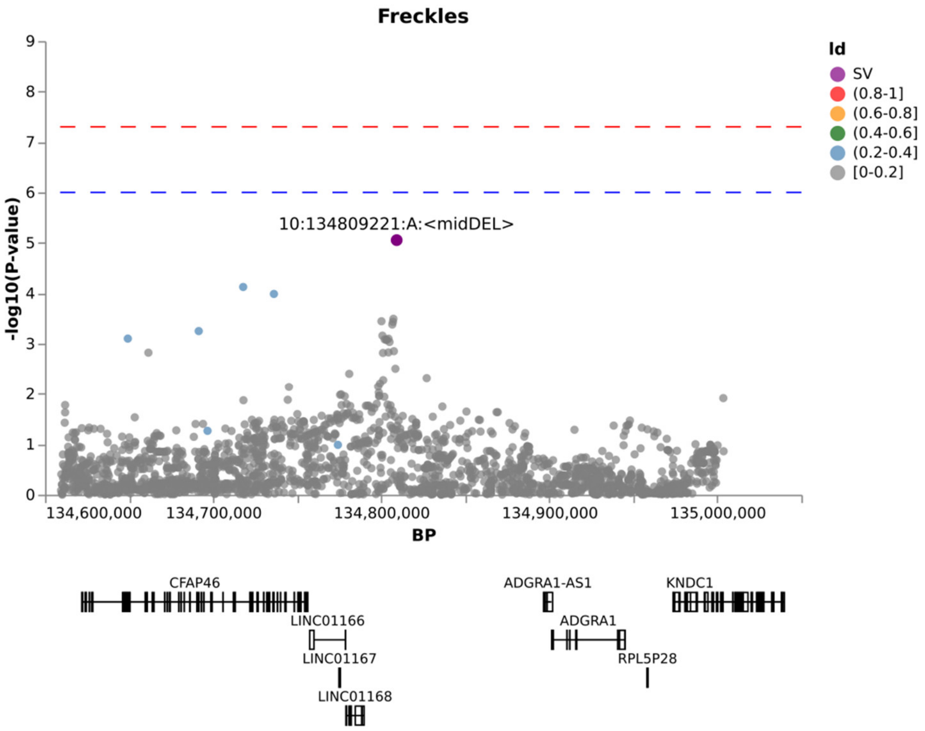

11:88964483:T:<DEL>

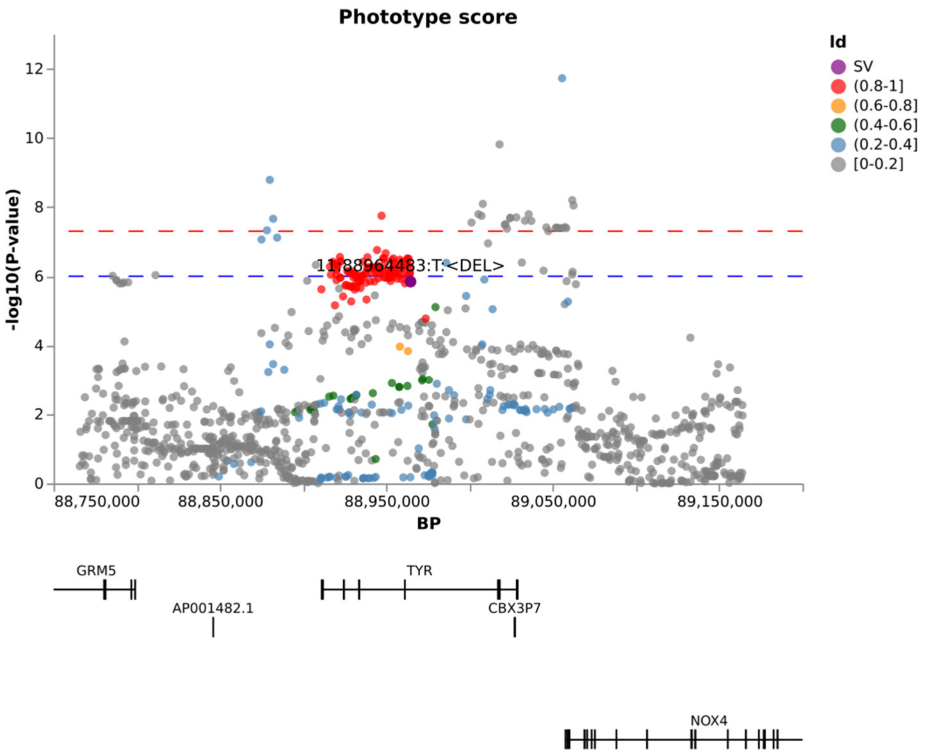

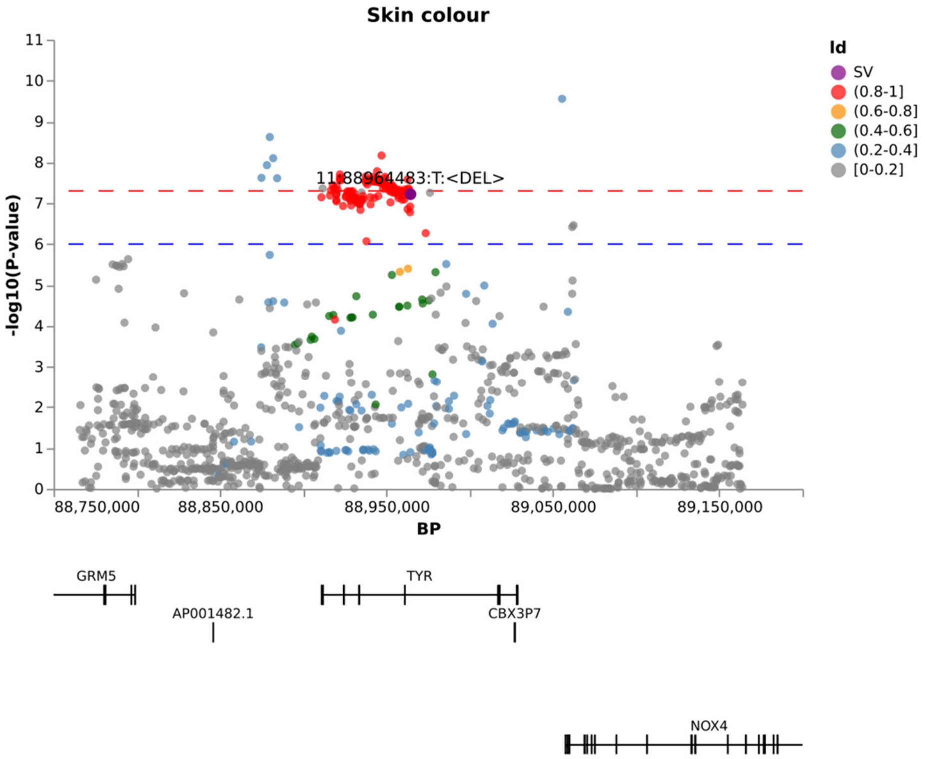

15:28268225:C:<midDEL>

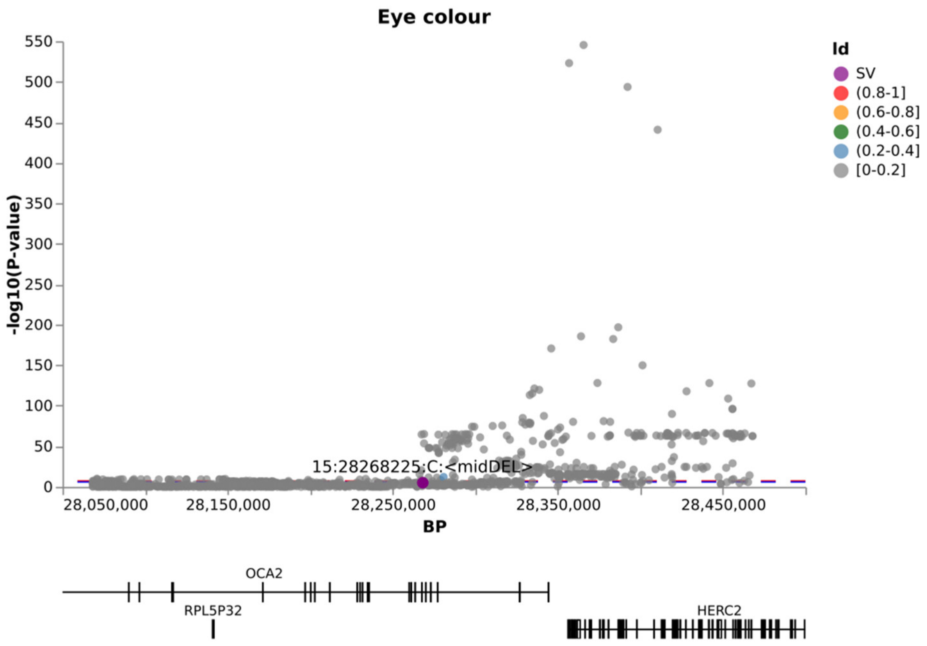

15:28473597:A:<DEL>

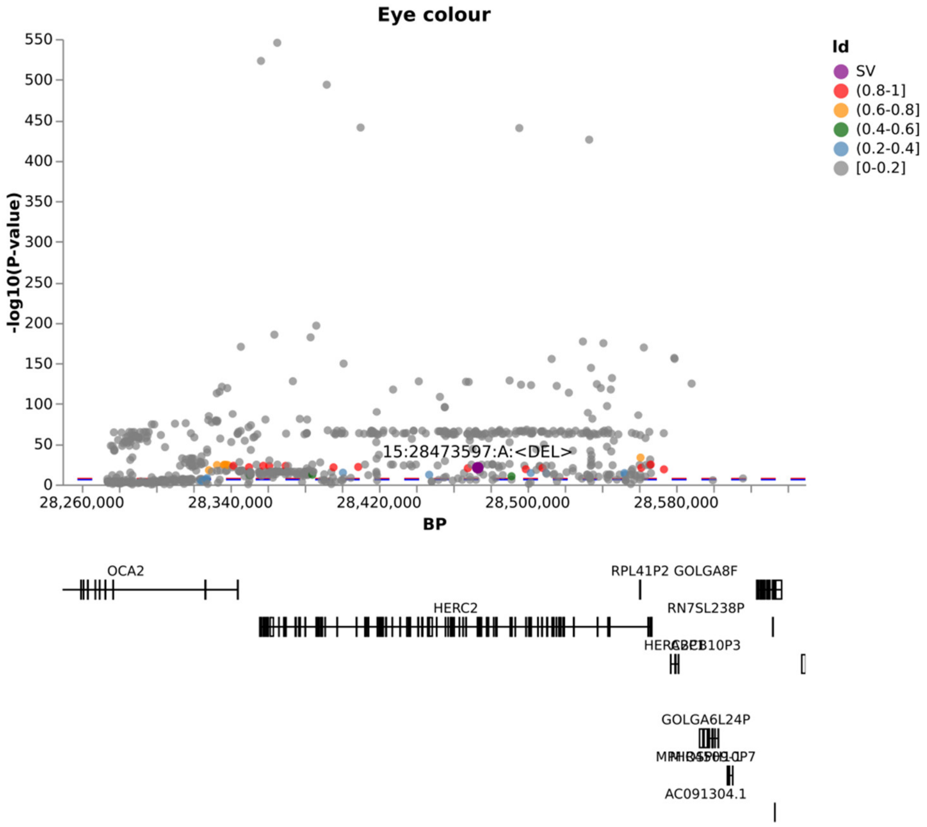

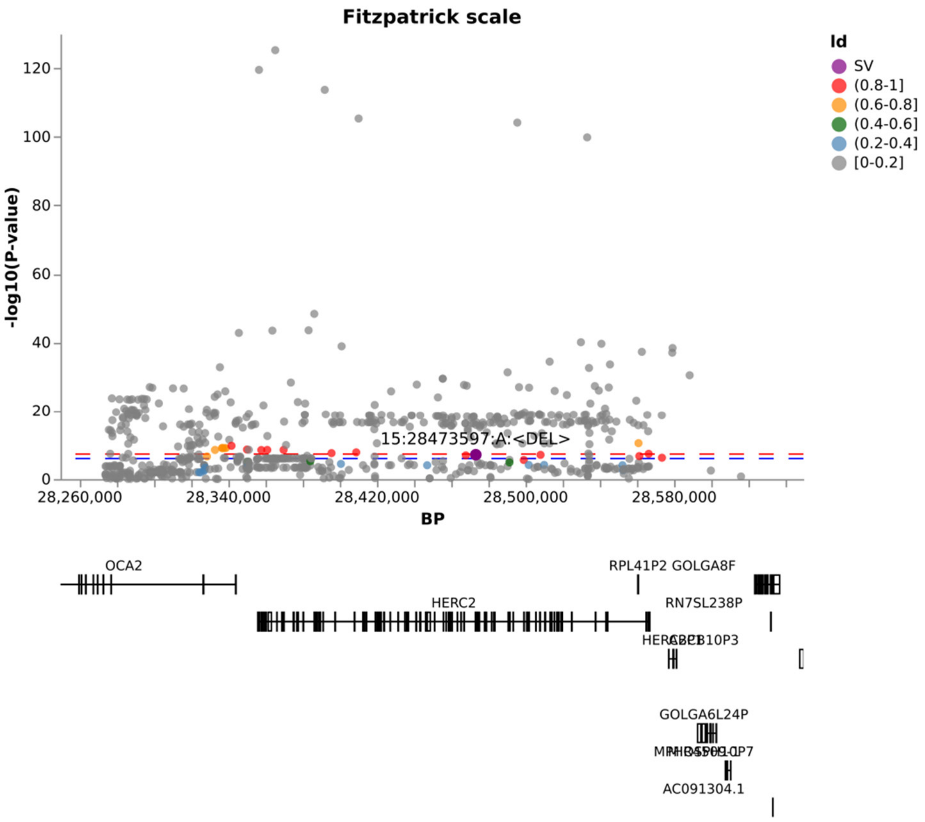
Fitzpatrick scale
-log10(P-value)
0
20
40
60
80
100
120
BP
28,260,000
28,340,000
28,420,000
28,500,000
28,580,000
15:28473597:A:<DEL>
ld
SV
(0.8-1]
(0.6-0.8]
(0.4-0.6]
(0.2-0.4]
[0-0.2]
OCA2
HERC2
RPL41P2 GOLGA8F
RN7SL238P
GOLGA6L24P
AC091304.1

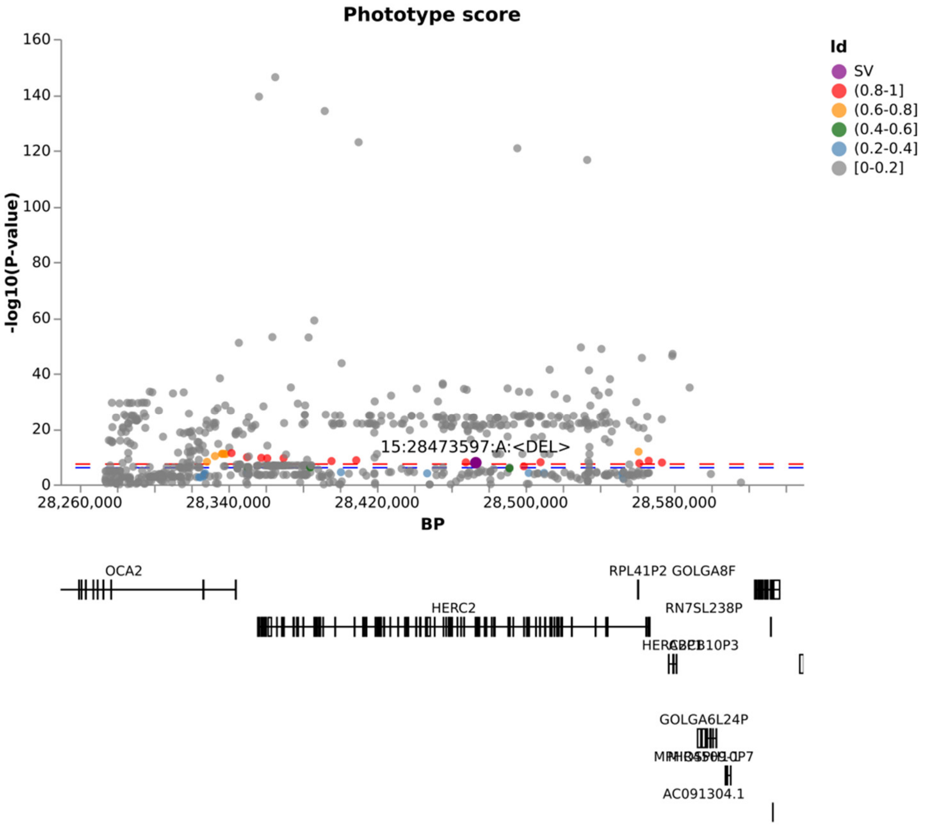
Phototype score
-log10(P-value)
0
20
40
60
80
100
120
140
160
BP
28,260,000
28,340,000
28,420,000
28,500,000
28,580,000
15:28473597:A:<DEL>
ld
SV
(0.8-1]
(0.6-0.8]
(0.4-0.6]
(0.2-0.4]
[0-0.2]
OCA2
HERC2
RPL41P2 GOLGA8F
RN7SL238P
GOLGA6L24P
AC091304.1

15:47924617:A:<DEL>

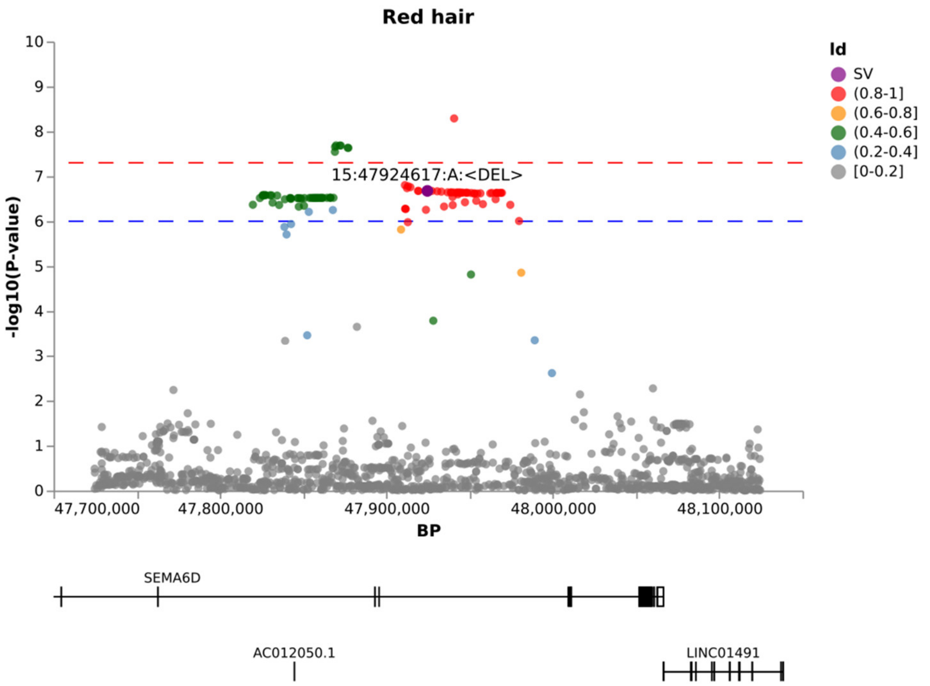

16:89657158:C:<midDEL>

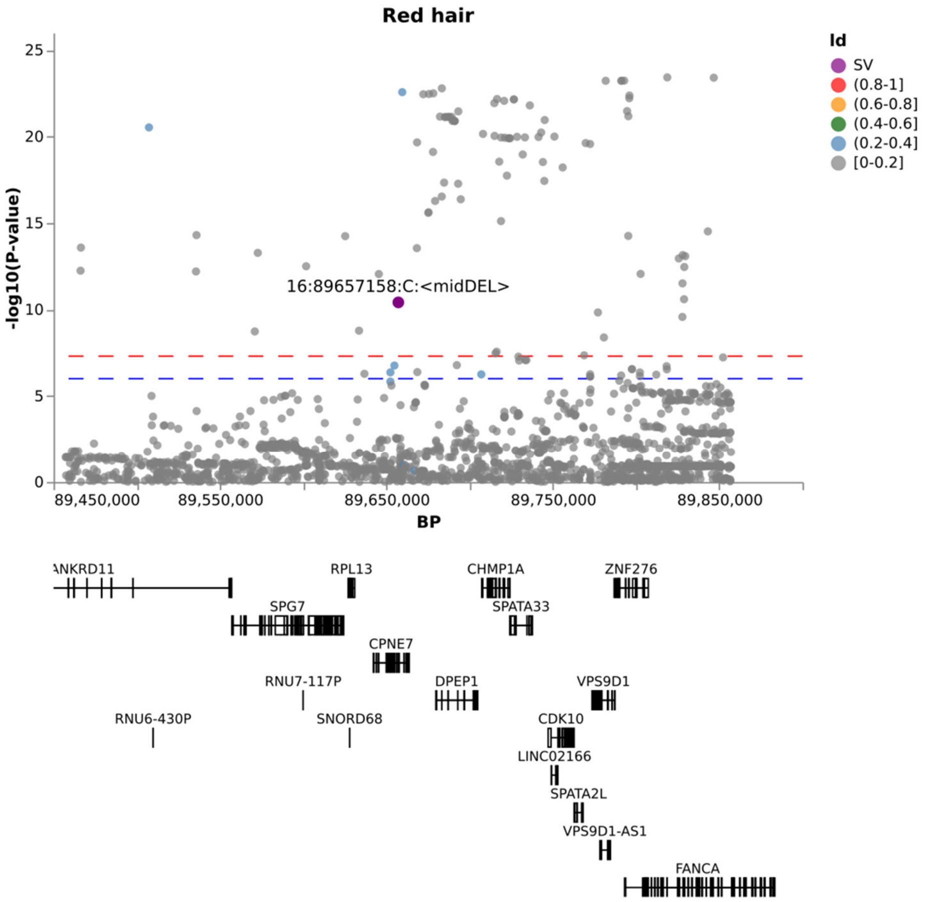

16:89715540:T:<DEL>

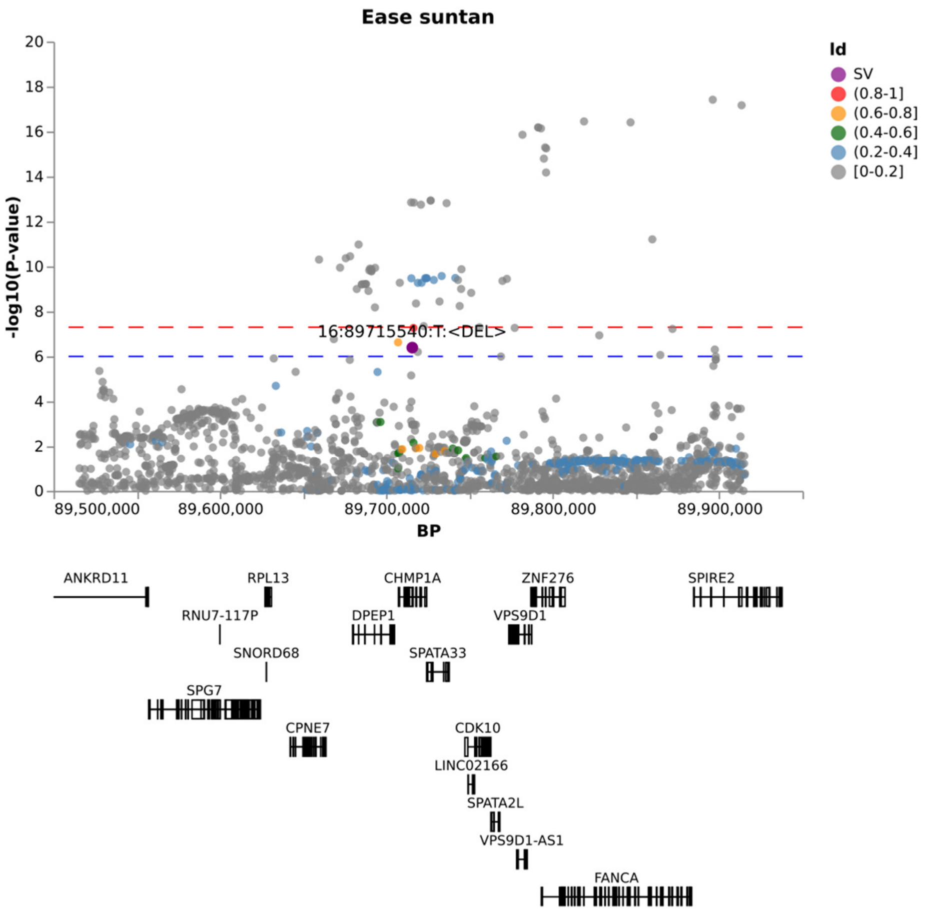

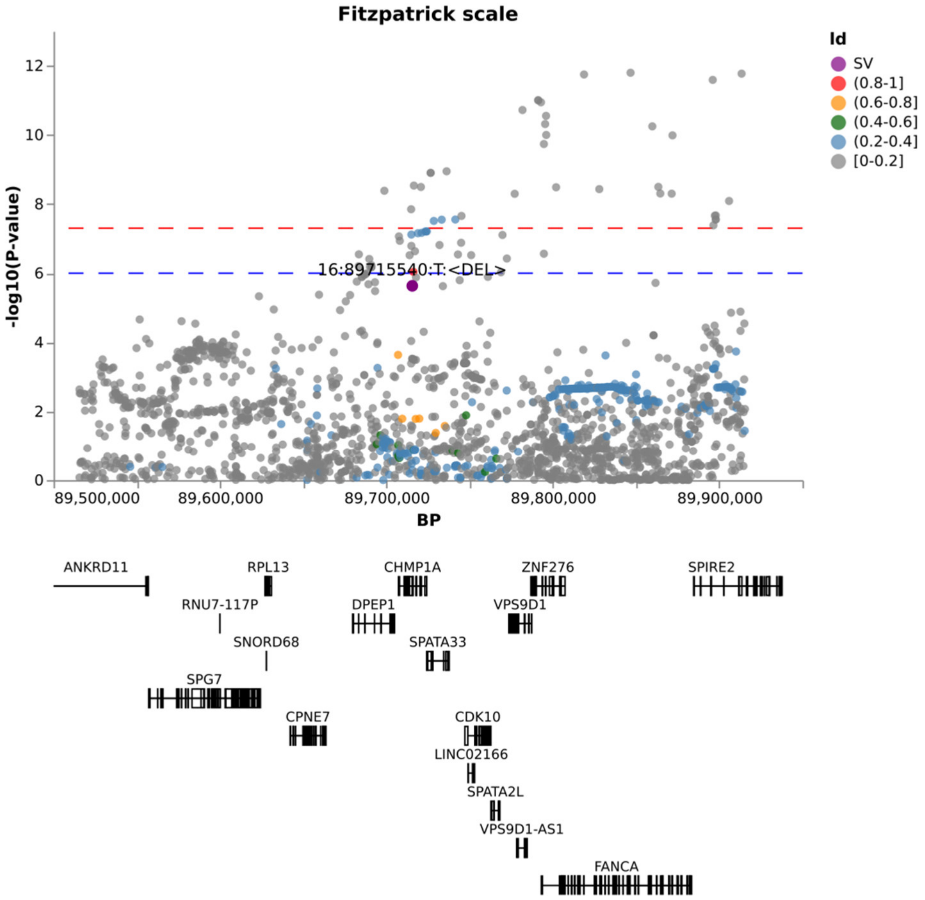
Fitzpatrick scale
ld
SV
(0.8-1]
(0.6-0.8]
(0.4-0.6]
(0.2-0.4]
[0-0.2]
-log10(P-value)
16:89715540:T:<DEL>
89,500,000
89,600,000
89,700,000
89,800,000
89,900,000
BP
ANKRD11
RPL13
CHMP1A
ZNF276
SPIRE2
RNU7-117P
DPEP1
VPS9D1
SNORD68
SPATA33
SPG7
CPNE7
CDK10
LINC02166
SPATA2L
VPS9D1-AS1
FANCA

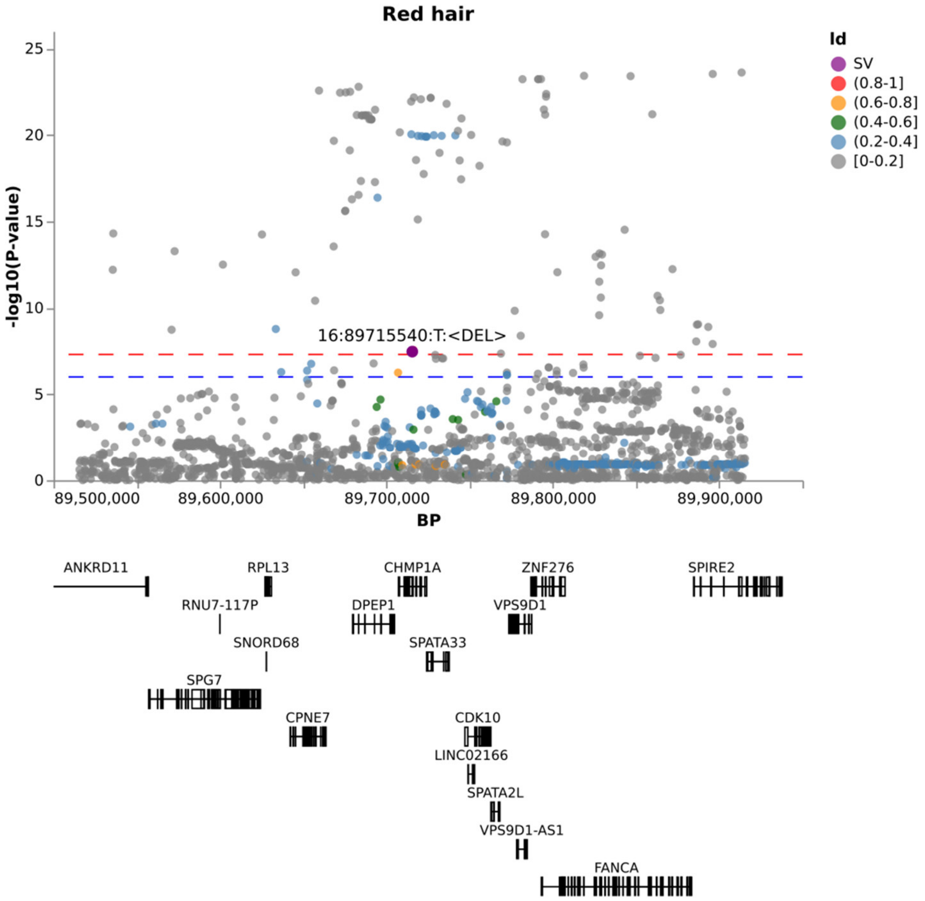
Red hair
ld
SV
(0.8-1]
(0.6-0.8]
(0.4-0.6]
(0.2-0.4]
[0-0.2]
-log10(P-value)
16:89715540:T:<DEL>
89,500,000
89,600,000
89,700,000
89,800,000
89,900,000
BP
ANKRD11
RPL13
CHMP1A
ZNF276
SPIRE2
RNU7-117P
DPEP1
VPS9D1
SNORD68
SPATA33
SPG7
CPNE7
CDK10
LINC02166
SPATA2L
VPS9D1-AS1
FANCA

16:89896057:G:<DEL>

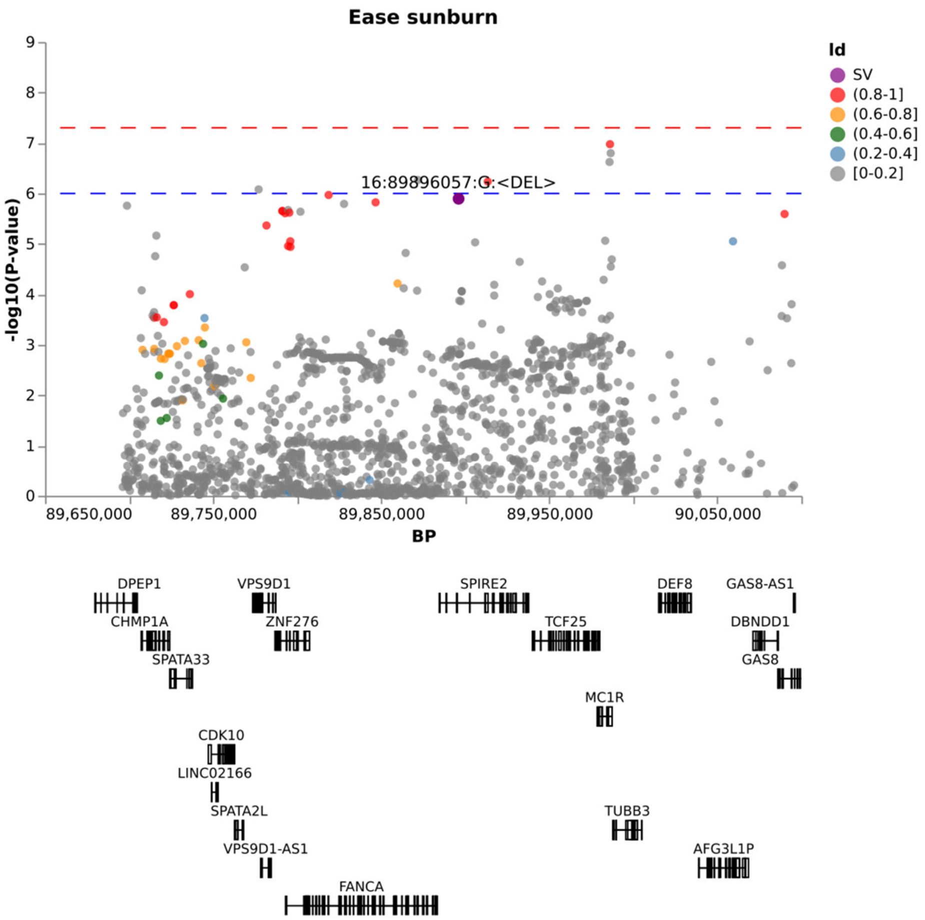

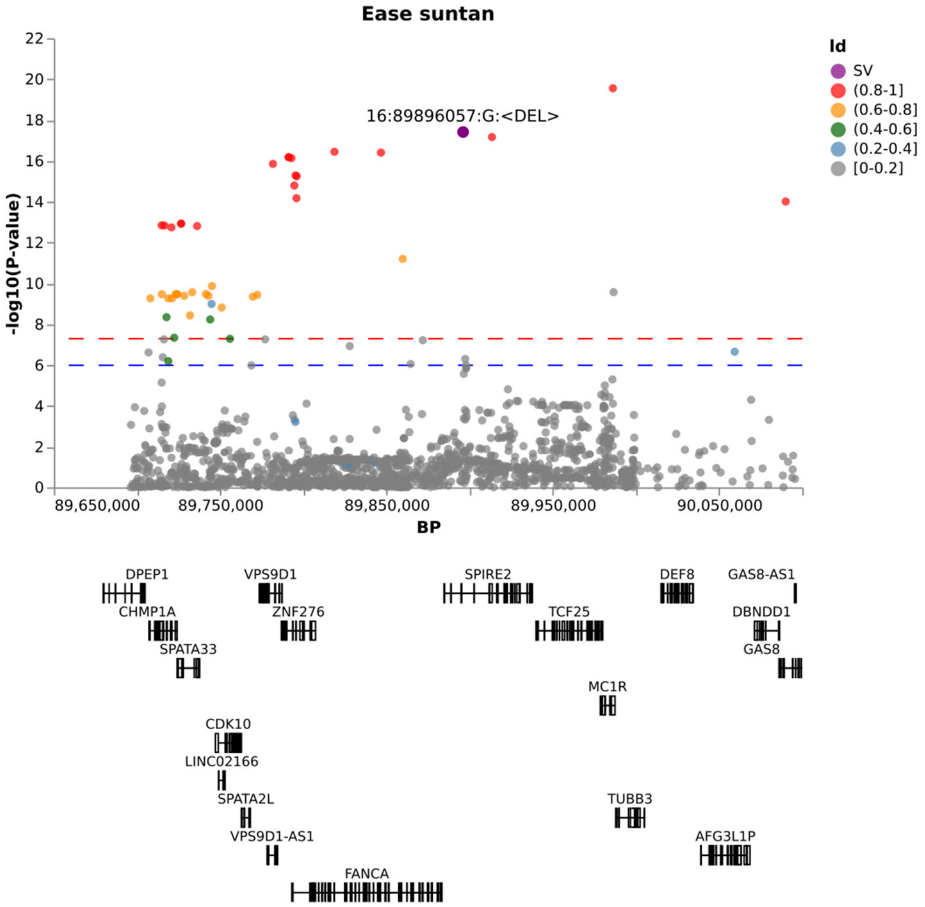
Ease suntan
-log10(P-value)
BP
89,650,000
89,750,000
89,850,000
89,950,000
90,050,000
16:89896057:G:<DEL>
ld
SV
(0.8-1]
(0.6-0.8]
(0.4-0.6]
(0.2-0.4]
[0-0.2]
DPEP1
VPS9D1
SPIRE2
DEF8
GAS8-AS1
CHMP1A
ZNF276
TCF25
DBNDD1
SPATA33
GAS8
MC1R
CDK10
LINC02166
SPATA2L
TUBB3
VPS9D1-AS1
AFG3L1P
FANCA

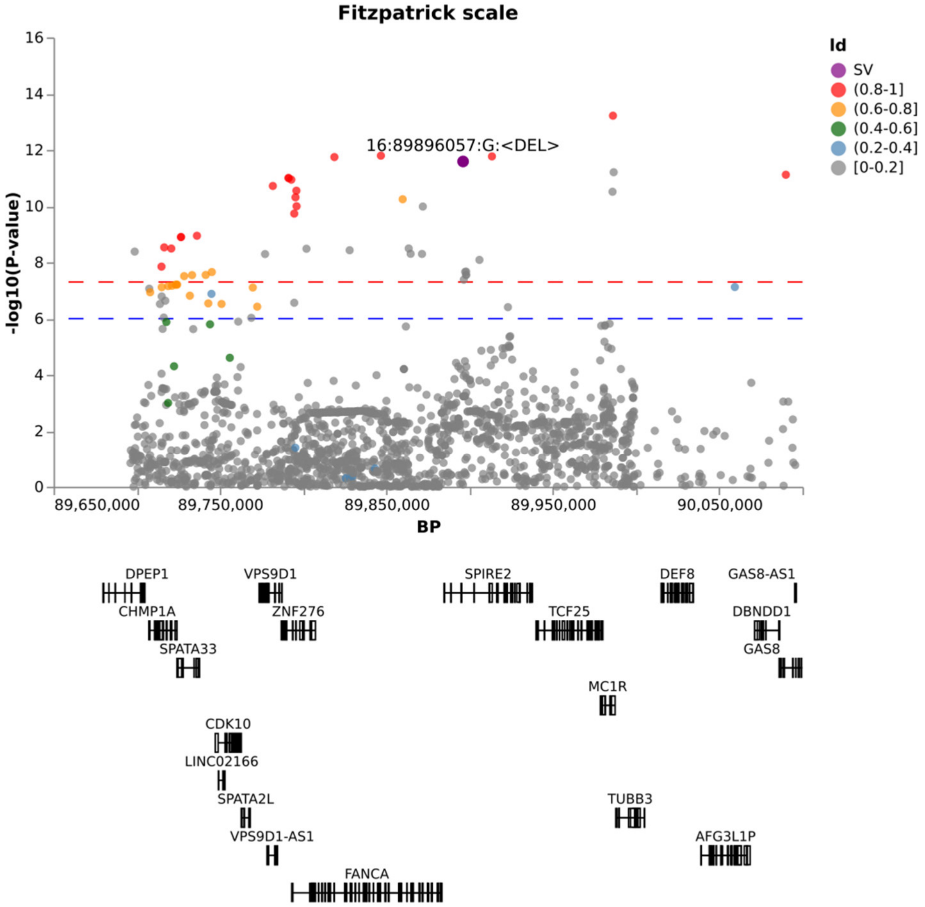
Fitzpatrick scale
-log10(P-value)
BP
89,650,000
89,750,000
89,850,000
89,950,000
90,050,000
16:89896057:G:<DEL>
ld
SV
(0.8-1]
(0.6-0.8]
(0.4-0.6]
(0.2-0.4]
[0-0.2]
DPEP1
VPS9D1
SPIRE2
DEF8
GAS8-AS1
CHMP1A
ZNF276
TCF25
DBNDD1
SPATA33
GAS8
MC1R
CDK10
LINC02166
SPATA2L
TUBB3
VPS9D1-AS1
AFG3L1P
FANCA

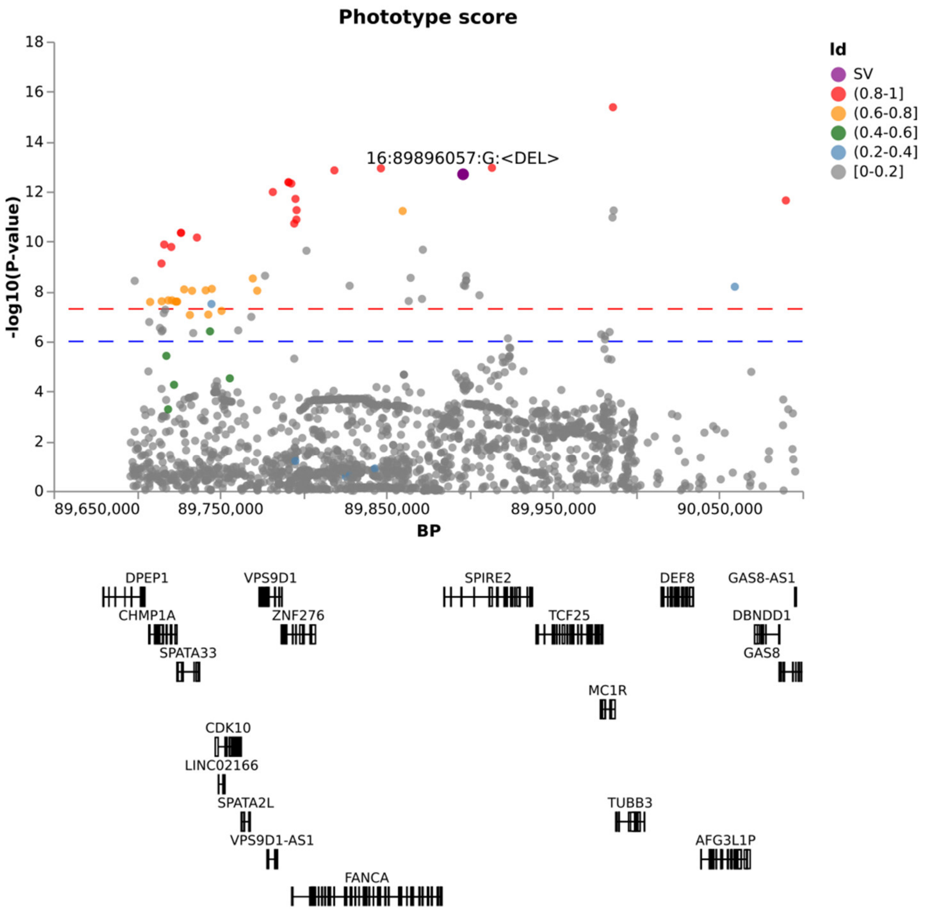
Phototype score
16:89896057:G:<DEL>
-log10(P-value)
BP
89,650,000
89,750,000
89,850,000
89,950,000
90,050,000
ld
SV
(0.8-1]
(0.6-0.8]
(0.4-0.6]
(0.2-0.4]
[0-0.2]
DPEP1
CHMP1A
SPATA33
CDK10
LINC02166
SPATA2L
VPS9D1-AS1
VPS9D1
ZNF276
FANCA
SPIRE2
TCF25
MC1R
TUBB3
DEF8
GAS8-AS1
DBNDD1
GAS8
AFG3L1P

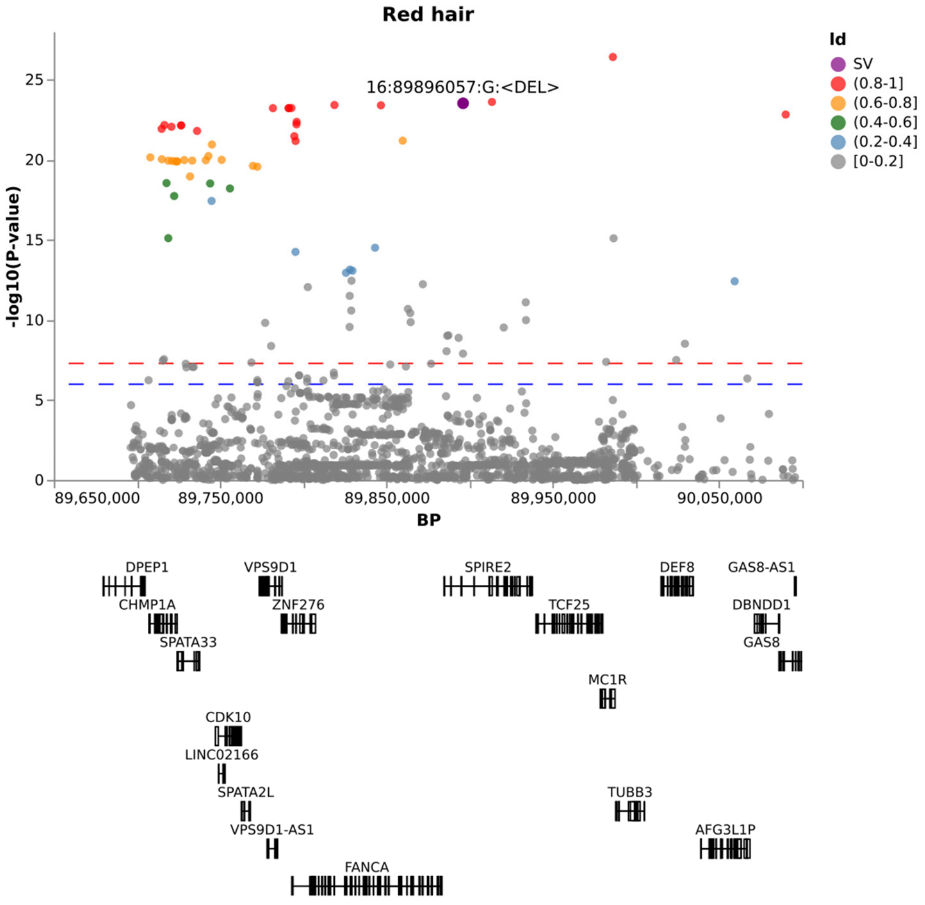
Red hair
16:89896057:G:<DEL>
-log10(P-value)
BP
89,650,000
89,750,000
89,850,000
89,950,000
90,050,000
ld
SV
(0.8-1]
(0.6-0.8]
(0.4-0.6]
(0.2-0.4]
[0-0.2]
DPEP1
CHMP1A
SPATA33
CDK10
LINC02166
SPATA2L
VPS9D1-AS1
VPS9D1
ZNF276
FANCA
SPIRE2
TCF25
MC1R
TUBB3
DEF8
GAS8-AS1
DBNDD1
GAS8
AFG3L1P

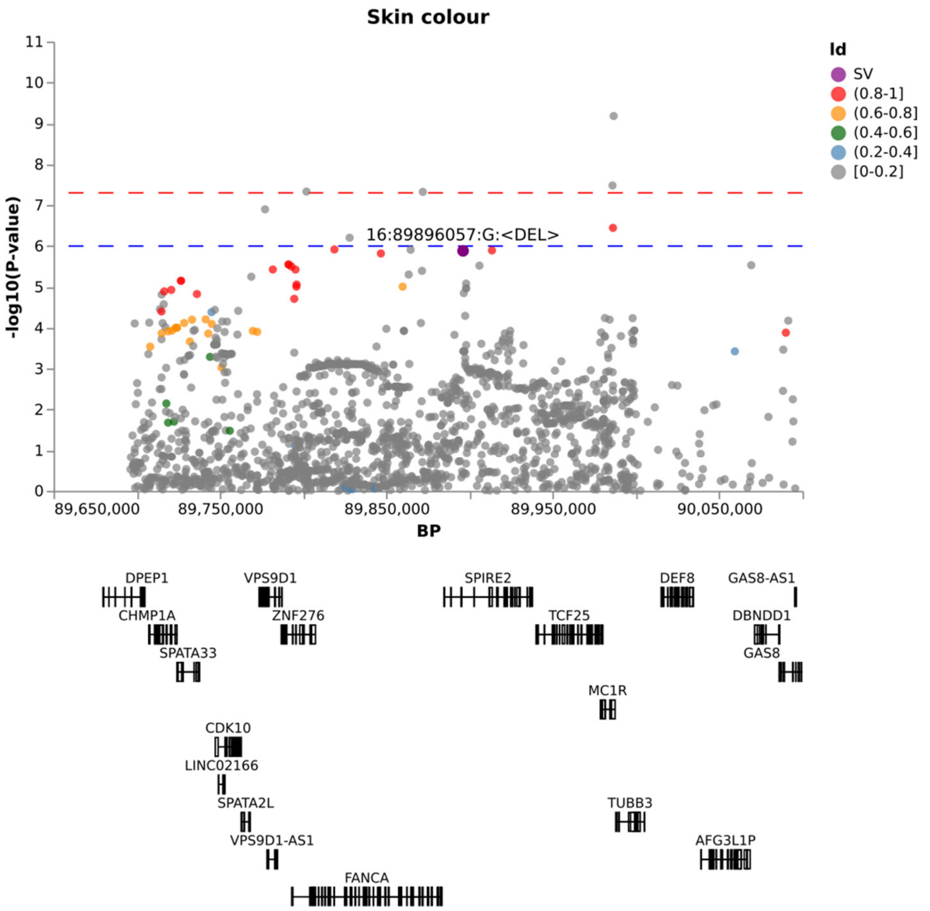

Skin colour
-log10(P-value)
BP
89,650,000
89,750,000
89,850,000
89,950,000
90,050,000
16:89896057:G:<DEL>
ld
SV
(0.8-1]
(0.6-0.8]
(0.4-0.6]
(0.2-0.4]
[0-0.2]
DPEP1
VPS9D1
SPIRE2
DEF8
GAS8-AS1
CHMP1A
ZNF276
TCF25
DBNDD1
SPATA33
GAS8
MC1R
CDK10
LINC02166
SPATA2L
TUBB3
VPS9D1-AS1
AFG3L1P
FANCA

16:90089999:G:<DEL>

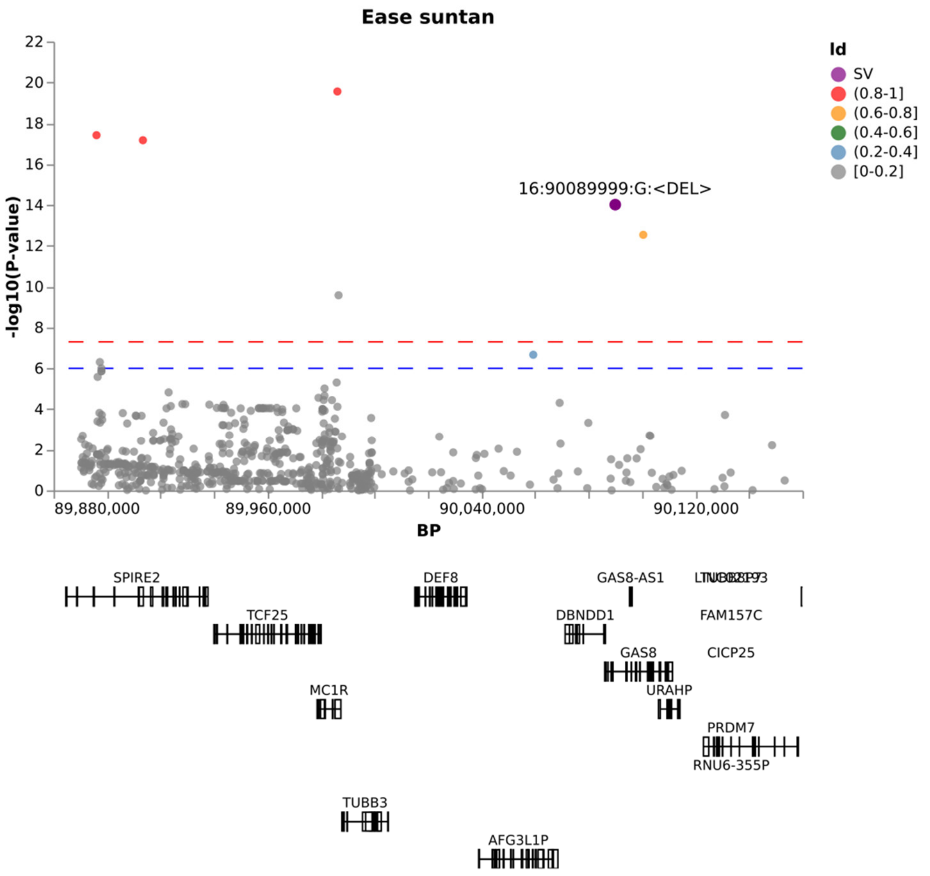

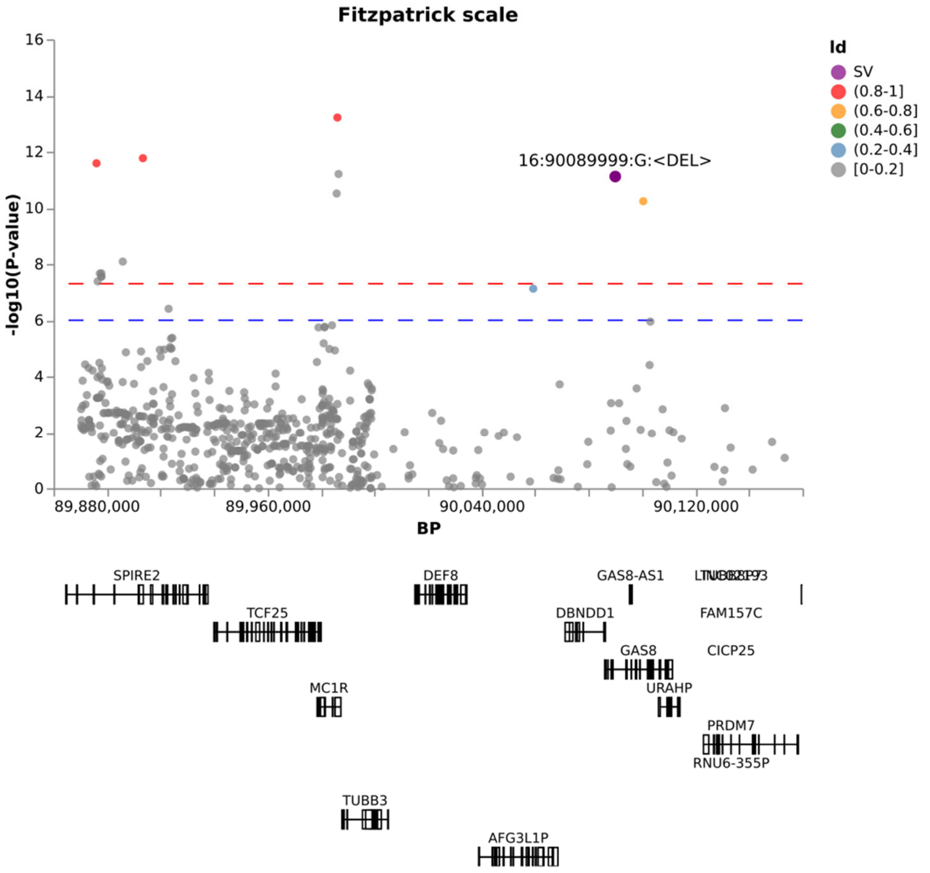

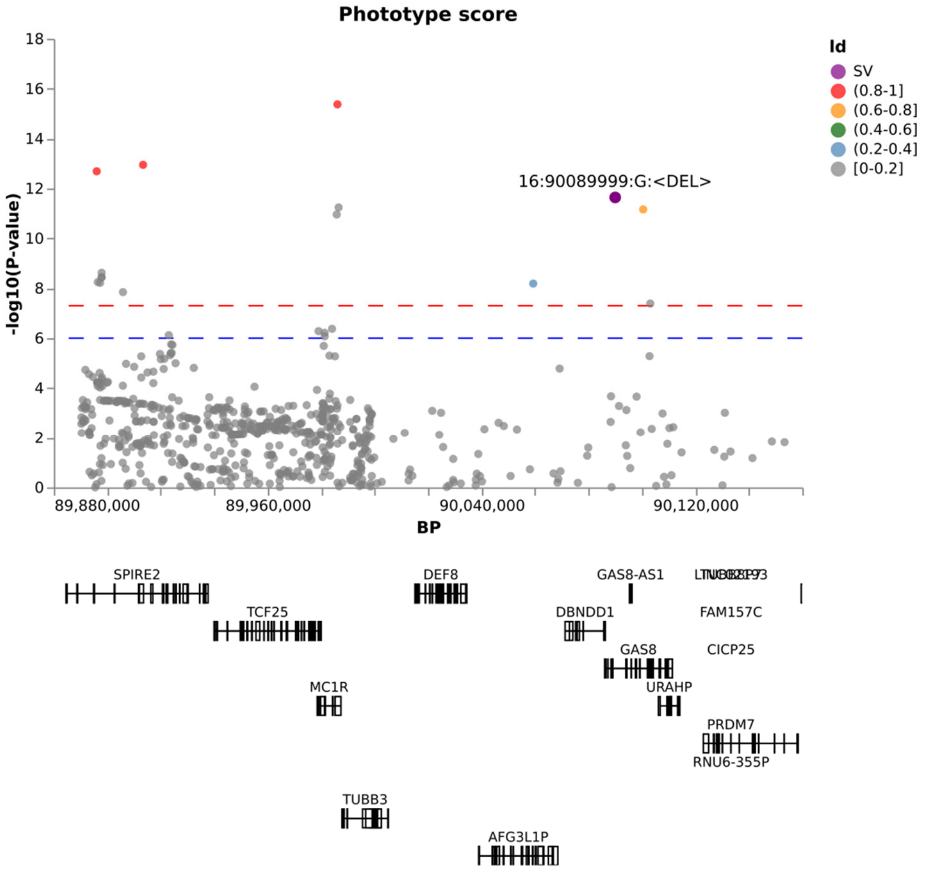
Phototype score
Id
SV
(0.8-1]
(0.6-0.8]
(0.4-0.6]
(0.2-0.4]
[0-0.2]
16:90089999:G:<DEL>
-log10(P-value)
BP
89,880,000
89,960,000
90,040,000
90,120,000
SPIRE2
TCF25
MC1R
TUBB3
DEF8
AFG3L1P
GAS8-AS1
DBNDD1
GAS8
URAHP
FAM157C
CICP25
PRDM7
RNU6-355P

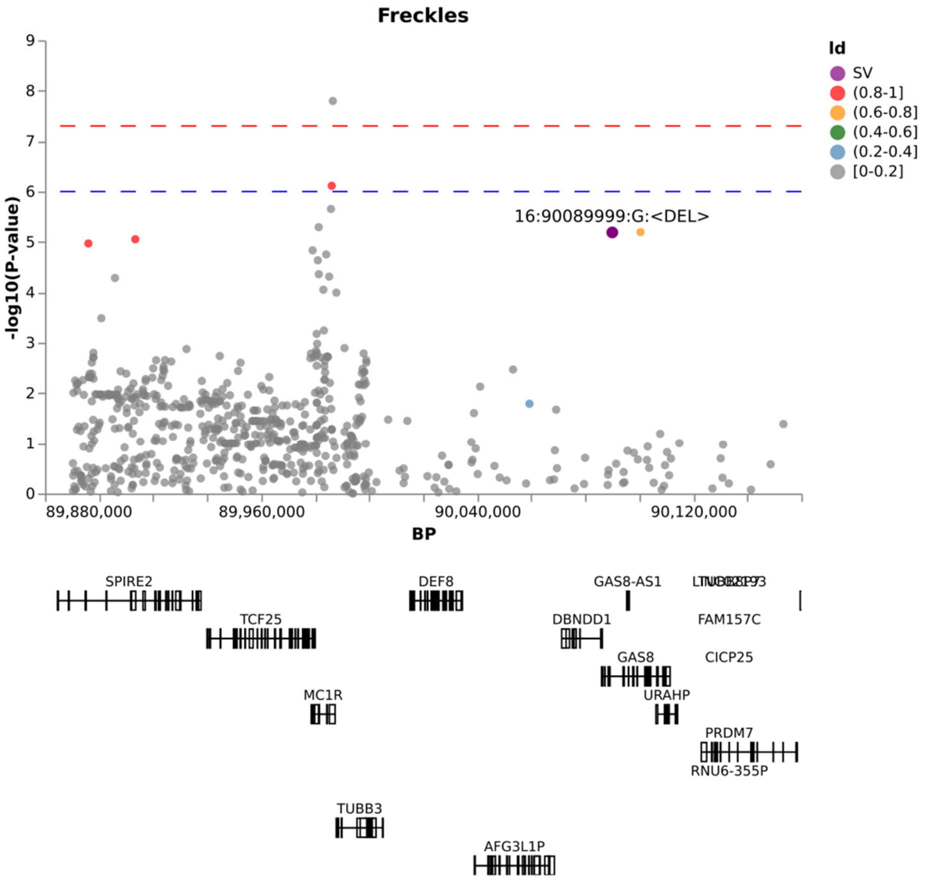
Freckles
Id
SV
(0.8-1]
(0.6-0.8]
(0.4-0.6]
(0.2-0.4]
[0-0.2]
16:90089999:G:<DEL>
-log10(P-value)
BP
89,880,000
89,960,000
90,040,000
90,120,000
SPIRE2
TCF25
MC1R
TUBB3
DEF8
AFG3L1P
GAS8-AS1
DBNDD1
GAS8
URAHP
FAM157C
CICP25
PRDM7
RNU6-355P

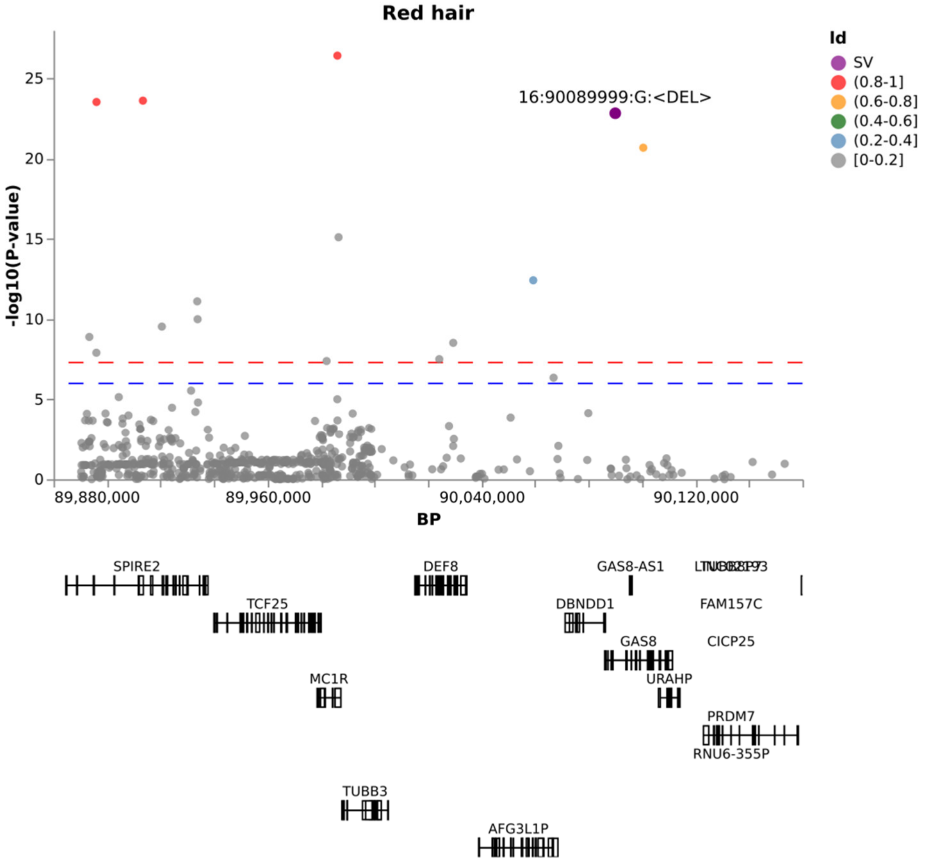
Red hair
Id
SV
(0.8-1]
(0.6-0.8]
(0.4-0.6]
(0.2-0.4]
[0-0.2]
16:90089999:G:<DEL>
-log10(P-value)
0
5
10
15
20
25
89,880,000
89,960,000
90,040,000
90,120,000
BP
SPIRE2
TCF25
MC1R
TUBB3
DEF8
AFG3L1P
GAS8-AS1
DBNDD1
GAS8
URAHP
FAM157C
CICP25
PRDM7
RNU6-355P

Supplementary Figure S7. Association of PRS of skin and sense organs diseases with pigmentary traits. Forest plot depicting the effect size of selected significant PRS (A) Glaucoma, (B) Psoriasis and (C) Essential hypertension. Upper plots, depicting the beta value and the 95% CI of discrete and continuous variables, and bottom plots, depicting the odds ratio and the 95% CI of binary variables, for each regression between PRS and pigmentary traits. X axis indicates the effect size for each pigmentary trait (Y axis). Additional information about beta, CI and p-value is shown.

A) Glaucoma

Glaucoma

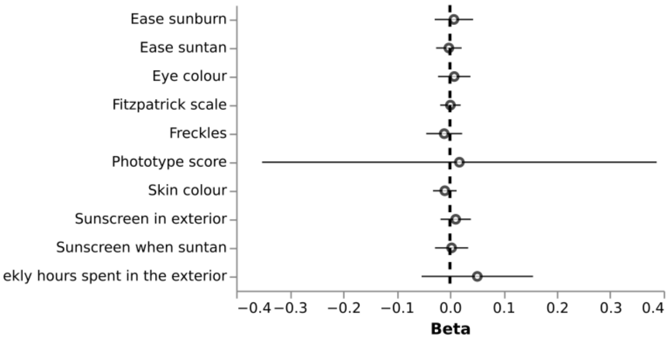

Beta [95% conf int]	p-value
0.01 [-0.03,0.04]	6.90e-1
-0.0 [-0.03,0.02]	8.64e-1
0.01 [-0.02,0.04]	6.07e-1
0.0 [-0.02,0.02]	9.31e-1
-0.01 [-0.04,0.02]	5.29e-1
0.02 [-0.35,0.39]	9.25e-1
-0.01 [0.01,-0.03]	3.91e-1
0.01 [-0.02,0.04]	4.59e-1
0.0 [-0.03,0.03]	8.48e-1
0.05 [-0.05,0.16]	3.36e-1

Glaucoma

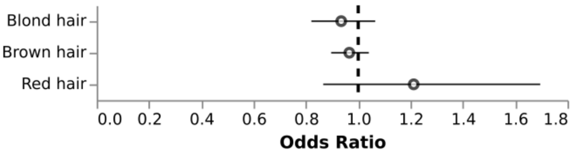

Odds Ratio [95% conf int]	p-value
0.93 [0.82,1.06]	3.11e-1
0.97 [0.9,1.04]	3.60e-1
1.21 [0.87,1.7]	2.62e-1

B) Psoriasis

Psoriasis

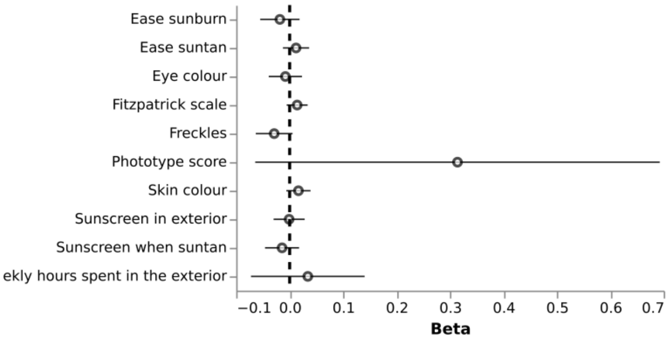

Beta [95% conf int]	p-value
-0.02 [-0.06,0.02]	3.30e-1
0.01 [-0.01,0.04]	3.38e-1
-0.01 [-0.04,0.02]	6.14e-1
0.01 [-0.01,0.03]	1.60e-1
-0.03 [-0.06,0.01]	9.92e-2
0.31 [-0.06,0.69]	1.04e-1
0.02 [0.04,-0.01]	1.54e-1
-0.0 [-0.03,0.03]	9.47e-1
-0.01 [-0.05,0.02]	3.81e-1
0.03 [-0.07,0.14]	5.31e-1

Psoriasis

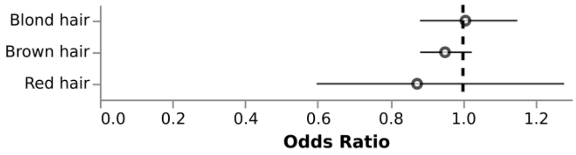

	Odds Ratio [95% conf int]	p-value
Blond hair	1.01 [0.88,1.15]	9.21e-1
Brown hair	0.95 [0.88,1.02]	1.83e-1
Red hair	0.87 [0.6,1.28]	4.87e-1

C) Essential hypertension

Essential hypertension

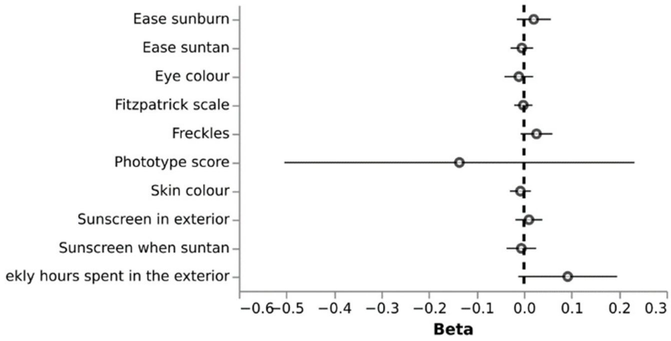

	Beta [95% conf int]	p-value
Ease sunburn	0.02 [-0.02,0.06]	2.59e-1
Ease suntan	-0.0 [-0.03,0.02]	7.14e-1
Eye colour	-0.01 [-0.04,0.02]	4.84e-1
Fitzpatrick scale	-0.0 [-0.02,0.02]	8.83e-1
Freckles	0.03 [-0.01,0.06]	1.23e-1
Phototype score	-0.14 [-0.5,0.23]	4.70e-1
Skin colour	-0.01 [0.01,-0.03]	5.09e-1
Sunscreen in exterior	0.01 [-0.02,0.04]	4.65e-1
Sunscreen when suntan	-0.01 [-0.04,0.03]	7.24e-1
ekly hours spent in the exterior	0.09 [-0.01,0.2]	8.29e-2

Essential hypertension

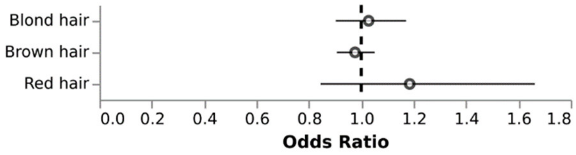

	Odds Ratio [95% conf int]	p-value
Blond hair	1.03 [0.9,1.17]	6.75e-1
Brown hair	0.98 [0.91,1.05]	5.22e-1
Red hair	1.18 [0.84,1.66]	3.26e-1
